# Complex Oxides under Simulated Electric Field: Determinants of Defect Polarization in *AB*O_3_ Perovskites

**DOI:** 10.1002/advs.202104476

**Published:** 2021-12-10

**Authors:** Yen‐Ting Chi, Krystyn J. Van Vliet, Mostafa Youssef, Bilge Yildiz

**Affiliations:** ^1^ Department of Materials Science & Engineering Massachusetts Institute of Technology Cambridge MA 02139 USA; ^2^ Department of Mechanical Engineering The American University in Cairo AUC Avenue, P.O. Box 74 New Cairo 11835 Egypt; ^3^ Department of Nuclear Science & Engineering Massachusetts Institute of Technology Cambridge MA 02139 USA

**Keywords:** density functional theory, perovskites, point defects, polarization, strain

## Abstract

Polarization of ionic and electronic defects in response to high electric fields plays an essential role in determining properties of materials in applications such as memristive devices. However, isolating the polarization response of individual defects has been challenging for both models and measurements. Here the authors quantify the nonlinear dielectric response of neutral oxygen vacancies, comprised of strongly localized electrons at an oxygen vacancy site, in perovskite oxides of the form *AB*O_3_. Their approach implements a computationally efficient local Hubbard *U* correction in density functional theory simulations. These calculations indicate that the electric dipole moment of this defect is correlated positively with the lattice volume, which they varied by elastic strain and by A‐site cation species. In addition, the dipole of the neutral oxygen vacancy under electric field increases with increasing reducibility of the B‐site cation. The predicted relationship among point defect polarization, mechanical strain, and transition metal chemistry provides insights for the properties of memristive materials and devices under high electric fields.

## Graphical abstract

A novel local Hubbard *U* method resolves challenges in simulating defected semi‐conducting oxides under electric field. This approach reveals that the oxygen vacancy dipole moment correlates positively with lattice volume and B‐site cation electronegativity. The predicted relationships among point defect polarization, mechanical strain, and transition metal chemistry provide insights for properties of memristive materials and devices under high electric fields.

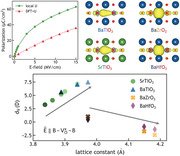

## 1 Introduction

Point defects such as oxygen vacancies play an important role in memristive devices.^[^ ^1^, ^2^, ^3^, ^4^ ^]^ Particularly in devices comprising thin films, these materials operate under high electric fields (on the order of megavolt per centimeter). Recent studies have shown that polarization of point defects under such high electric fields can strongly impact defect formation,^[^ ^5^ ^]^ transport,^[^ ^6^ ^]^ and distribution, as well as dielectric response,^[^ ^7^ ^]^ which are integral to device performance. In memristive devices, the formation and migration of oxygen vacancies under strong electric fields directly facilitate conductive filament growth,^[^ ^8^, ^9^ ^]^ which in turn governs device switching speed, state stability, and durability.^[^ ^1^, ^8^ ^]^ While earlier works have demonstrated the significance of defect polarization to device performance under high external electric field, it is important to further identify and quantify the impact of factors controlling field‐dependent polarization of point defects, and this knowledge can facilitate materials design for devices with targeted functionalities.^[^ ^8^, ^10^ ^]^

However, decoupling the factors driving defect polarization in functional oxides has been non‐trivial. Using state‐of‐the‐art aberration‐corrected scanning transmission electron microscopy (AC‐STEM), Gao et al.^[^ ^11^ ^]^ demonstrated direct imaging of local charge density in a SrTiO_3_‐BiFeO_3_ heterojunction with sub‐Angstrom resolution. Kumar et al.^[^ ^12^ ^]^ have also shown nanoscale polarization mapping in Pb‐based ferroelectrics by combining annular dark‐field and integrated differential phase contrast imaging.^[^ ^13^, ^14^ ^]^ Nevertheless, such experimental techniques have not been demonstrated in resolving the behavior of single isolated point defects, and are not yet practical to implement over a broad range of compositions and conditions. This motivates the development of reliable computational approaches to predict and quantify the factors controlling defect polarizability more directly and more efficiently than possible with present experiments.

Computationally, density functional theory (DFT) has known drawbacks in simulating defected semiconducting systems under an electric field accurately. For defects with an in‐gap state in semiconducting systems, pure DFT gives rise to an incorrect metallic solution^[^ ^15^, ^16^ ^]^ such that polarization calculations with the Berry phase approach^[^ ^17^, ^18^ ^]^ are inapplicable. DFT augmented with Hubbard *U* terms (DFT + *U*) is necessary to ensure localized defect states in a semiconducting system, but this approach significantly underestimates the materials’ polarization response under an electric field.^[^ ^5^, ^19^, ^20^ ^]^ Although hybrid functionals can address these challenges, such a method is currently not implemented with the Berry phase approach to perform ion relaxation under electric field. For the Quantum Espresso package 6.4.1^[^ ^21^, ^22^ ^]^ used in this study, the iteration processes of these two approaches would interfere with each other, resulting in questionable results for a self‐consistent single‐shot calculation, and fail to converge for ionic relaxation. Therefore, we use a “local Hubbard *U*” method to circumvent these challenges in this work.

Here, we deconvolute the role of two factors that we hypothesize to affect the polarizability of a point defect in complex oxides: lattice volume and reducibility of ions surrounding the point defect. Specifically, we assessed the polarizability of the neutral oxygen vacancy, characterized by two electrons trapped in the vacant site (color center, singlet state) in an *AB*O_3_ cubic perovskite oxide under electric field (see Section S1, Supporting Information, for defect magnetic configuration comparison). First, we resolved the defect dipole moment as a function of lattice volume, tuned by elastic strain in SrTiO_3_ or by changing the A‐site cation species (*A* = Sr or Ba). BaTiO_3_ has a larger A‐site cation and lattice volume compared to SrTiO_3_, otherwise maintaining the same transition metal and reduction–oxidation (redox) chemistry. Second, we resolved the role of reducibility of the B‐site transition metal cation surrounding the defect by varying the B‐site cation in Ba*B*O_3_ (*B* = Ti, Zr, or Hf) while keeping the same crystallographic symmetry and the same lattice volume (when needed) by applying hydrostatic strain. It is worth noting that the structural ground states for SrTiO_3_, BaZrO_3_, and BzHfO_3_ are already in the cubic phase under room temperature,^[^ ^23^, ^24^ ^]^ and selecting cubic BaTiO_3_ (high temperature phase) is necessary to facilitate a consistent comparison under the same crystallographic symmetry. The coupling of the defect polarization and the ferroelectric BaTiO_3_ phase is beyond the scope of the current study, but could be of interest in further work.

Let us consider the rationale for why these two factors could each influence point defect polarizability. Volumetric changes in an *AB*O_3_ crystal can be introduced via applied mechanical strain, or by composition (i.e., varying the A‐site cation at zero strain). Increasing free volume around a point defect alters the potential energy landscape around the defect, reducing the electronic interaction of that defect with its surroundings.^[^ ^25^, ^26^ ^]^ This means that the increased free volume around a neutral oxygen vacancy studied here reduces the constraints on the electron density occupying that volume, thereby making it easier for the trapped electrons to polarize under an electric field. We have also shown previously that lattice volume increase due to cation size among several simple binary oxides affects the polarizability of oxygen vacancies.^[^ ^5^ ^]^

Reducibility of transition metal ions surrounding the neutral oxygen vacancy can also play an important role in affecting the polarizability of the trapped electrons at the vacant site. By reducibility, we mean the ease of reduction of the cation by gaining a localized electron and formally changing its oxidation state. This is related to the energy levels of the electron defect state projected on that cation. It is possible to calculate these reduction–oxidation (redox) levels for each compound and defect type. However, in the spirit of establishing a simpler descriptor, we adopt electronegativity of the neutral transition metal to represent the reducibility of its charged cation. For metal elements of high electronegativity, we expect the corresponding ions should attract electrons more easily than those of metals with lower electronegativity. We can justify this hypothesis also by observing that the previously reported polarization of Bi*M*O_3_ (*M* = Al, Ga, Fe)^[^ ^27^, ^28^, ^29^ ^]^ correlates positively with the electronegativity of the B‐site cation. The polarization of the lone‐pair electrons on bismuth share some similarities with the polarization of the two trapped electrons in the oxygen vacancy treated in this work; they are both associated with local electronic states, the spatial distribution of which depends on the species of nearby ions.

## 2 Results

### 2.1 Modeling Defect Polarization under Electric Field Using a Local Hubbard *U* Method

**Figure 1:**
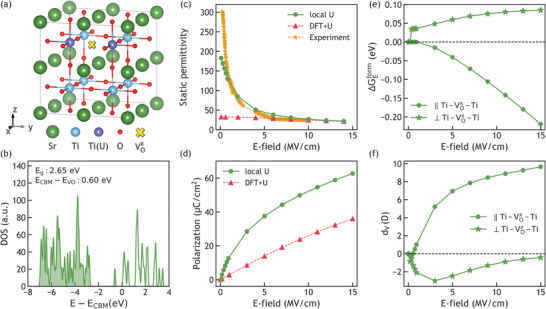
Density of states, dielectric properties and defect formation energy predicted for neutral oxygen vacancy $V_{O}^{x}$ in SrTiO_3_, using density functional theory with local Hubbard *U*. a) A 2 × 2 × 2 SrTiO_3_ super cell with $V_{O}^{x}$. Green, light blue, and red spheres represent Sr, Ti, and O ions, respectively. Dark blue spheres indicate the two Ti ions with local Hubbard *U*, adjacent to the trapped electrons in the oxygen vacancy site represented by a yellow cross. All crystals in this work were visualized by VESTA.^[^ ^41^ ^]^ b) Density of states for defected SrTiO_3_ under 3 MV cm^−1^ with shaded area representing occupied states. *E*_g_ and *E*_CBM_ are the band gap and the minimum energy of conduction band, respectively. *E*_CBM_ is set to be zero. Static dielectric permittivity (c) and polarization (d) of defected SrTiO_3_ with respect to applied electric field parallel to the $Ti^{4+}-V_{O}^{x}-Ti^{4+}$ chain direction, from 0 to 15 MV cm^−1^, using the local Hubbard *U* method (green circles), standard DFT + *U* method (red triangles), and the experimental values (orange stars). The non‐linear polarization predicted by local Hubbard *U* better reflects the high static permittivity of SrTiO_3_ under low field. Comparison of relative electric Gibbs free energy of formation of $V_{O}^{x}$ (e), and the vacancy dipole *d_V_* in units of Debye (f) with respect to applied field parallel to (circles) and perpendicular to (stars) the $Ti^{4+}-V_{O}^{x}-Ti^{4+}$ chain.

To address the challenges in studying the polarization of defective semiconducting oxides using DFT, we have adopted an approach based on the previously reported self‐consistent, site‐dependent DFT + *U* method.^[^ ^30^, ^31^ ^]^ This approach applies different Hubbard *U* magnitudes on different lattice sites in a defected system, especially for the sites closest to the defect. In our work, to minimize the potentially detrimental effect of Hubbard *U* on the polarization properties, we applied the Hubbard *U* correction only on the two Ti ions adjacent to the oxygen vacancy site to ensure charge localization, and we termed this approach the “local Hubbard *U*”. Choosing a very low magnitude of *U* (1 or 2 eV) would not achieve enough electron localization on the defect site, as confirmed by hybrid functionals.^[^ ^32^, ^33^ ^]^ A very high magnitude (>5 eV), on the other hand, would result in unphysical over localization. We tested *U* = 3, 4, and 5 eV and chose to implement *U* = 5 eV in our following study, since this magnitude resulted in consistent results between a 3 × 3 × 3 supercell and a 2 × 2 × 2 supercell. However, all magnitudes of *U* considered led to similar trends albeit with different magnitudes. Full methodology details are provided in Sections S1 and S 2, Supporting Information, while the framework and definitions are summarized here in **Figure** **1**. As shown in Figure 1a, we constructed a 2 × 2 × 2 SrTiO_3_ supercell with one neutral oxygen vacancy ($V_{O}^{x}$ with Kroger–Vink notation). We applied a Hubbard *U* correction^[^ ^34^ ^]^ of 5.0 eV only to the *d*‐states of the two Ti ions neighboring the oxygen vacancy, depicted as dark blue spheres. Figure 1b shows the density of states, illustrating that the local Hubbard *U* is sufficient to localize the two electrons in the defect site leading to a correct in‐gap electronic state under the electric field. At the same time, this approach together with the modern theory of polarization^[^ ^18^, ^35^ ^]^ yields a static permittivity of the overall defective crystal close to the experimental value.^[^ ^36^, ^37^ ^]^ Comparisons of static permittivity and polarization results arising from the standard Hubbard *U* and the local Hubbard *U* methods are shown in Figures 1c and 1d, respectively. The non‐linear polarization under low electric field predicted with the local Hubbard *U* better reflects the high static permittivity for SrTiO_3_ under low field as shown in Figure 1c), where the difference was attributed to slightly different lattice constants obtained by DFT and experiment. Using the local Hubbard method with the experimentally determined lattice constant of SrTiO_3_, our predicted static permittivity matches very well with the experimental static permittivity values^[^ ^37^ ^]^ under all fields (see Section S1, Supporting Information). Note that the bandgap of SrTiO_3_ obtained with the local Hubbard *U* method under zero field (2.54 eV) underestimates the experimental value (3.2 eV^[^ ^38^ ^]^), which we attributed to the intentional lack of a global Hubbard *U* correction on all the other Ti and O ions in our simulation. Despite the inaccurate bandgap, we believe our method provides the best compromise between defect and dielectric properties in semiconducting oxides with the current simulation package capabilities.

Having established a method to study defected SrTiO_3_ under electric field, we recall the definition of electric Gibbs free energy of formation $G_{E}^{\mathrm{form}}$ for a neutral oxygen vacancy^[^ ^5^ ^]^ $V_{O}^{x}$:

(1) $$G_{E}^{\mathrm{form}}=\left(U^{\mathrm{def}}-U^{\mathrm{perf}}+\mu_{O}\right)\;-V\overset{⇀}{E}\cdot \left({\overset{⇀}{P}}^{\mathrm{def}}-{\overset{⇀}{P}}^{\mathrm{perf}}\right)$$

where *U* and *P* are the internal energy and polarization of the defect‐free (perf) or defected (def) SrTiO_3_ under electric field, respectively; *μ*_O_ is the chemical potential of oxygen; *V* is the volume of the supercell; and $\overset{⇀}{E}$ is the electric field. The net dipole moment in the system arising from the introduction of $V_{o}^{x}$ into the system *d_V_* is given through the last term in Equation (1):

(2) $$d_{V}=V\cdot \left({\overset{⇀}{P}}^{\mathrm{def}}-{\overset{⇀}{P}}^{\mathrm{perf}}\right)$$

We will call this quantity the oxygen vacancy dipole moment, although with such definition this is not the dipole moment locally calculated at the vacant site. Instead, it is the difference between the dipole moments of the defected and perfect crystals due to the creation of one oxygen vacancy. By performing site‐decomposed dipole moment analysis using Wannier functions,^[^ ^39^, ^40^ ^]^ we found that the two electrons at the oxygen vacant site in SrTiO_3_ contribute largely to *d_V_* (see Section S5, Supporting Information), consistent with our previous work on binary oxides.^[^ ^5^ ^]^

Figure 1e shows $\Delta G_{E}^{\mathrm{form}}$ as a function of electric field, and it is defined as the difference in $V_{O}^{x}\;$formation energy between finite and zero field conditions. When the electric field was applied parallel to the $Ti^{4+}-V_{O}^{x}-Ti^{4+}$ chain, $\Delta G_{E}^{\mathrm{form}}$decreased with increasing field magnitude. Note that each oxide ion is twofold coordinated by Ti in SrTiO_3_. In contrast, when the field was applied perpendicular to the $Ti^{4+}-V_{O}^{x}-Ti^{4+}$ chain, $\Delta G_{E}^{\mathrm{form}}$increased with increasing field. As such we conclude that the electric field breaks the symmetry of the crystal and creates two populations of oxygen sites; those with $Ti^{4+}-V_{O}^{x}-Ti^{4+}$ chains aligned parallel to the field, and those with chains perpendicular to the field. At zero field, both populations are equally likely to host a vacancy. Under a finite field, the chain parallel to the field (1/3 of the total oxygen population) is more likely to host a vacancy. $\Delta G_{E}^{\mathrm{form}}$ is determined mainly by the *d_V_* defined above, and to a much lesser extent by the change in the internal energy (see first term in Equation (1), with details in Section S2, Supporting Information). Figure 1f shows the effect of the electric field direction on *d_V_* in SrTiO_3_, exhibiting a sign dependence of *d_V_* on the field direction with respect to the $Ti^{4+}-V_{O}^{x}-Ti^{4+}$ chain. With field applied parallel to <100> in cubic SrTiO_3_, the trapped electrons would either be polarized toward one of the neighboring Ti ions (field parallel to the $Ti^{4+}-V_{O}^{x}-Ti^{4+},$ which is the case for 1/3 of the oxygen sites); or toward the space between the nearest Sr ions (field perpendicular to the $Ti^{4+}-V_{O}^{x}-Ti^{4+},$ which is the case for 2/3 of the oxygen sites). Positive *d_V_* and decreasing $\Delta G_{E}^{\mathrm{form}}$ are consistent with the favored polarization direction being parallel to the $Ti^{4+}-V_{O}^{x}-Ti^{4+}$ chain. To focus the discussion on the dominant vacancy population, the results below are concerned primarily with the electric field applied parallel to the $Ti^{4+}-V_{O}^{x}-Ti^{4+}$ chain.

### 2.2 Lattice Volume Effect on Oxygen Vacancy Dipole Moment, *d_V_*

Having adapted a reliable computational approach to accurately predict the electronic and ionic structure, static permittivity, and polarization in a semiconducting perovskite, we sought to assess the role of the two hypothesized factors: lattice space available for the defect, and electronegativity of the cations closest to the defect site. To test the former, we tuned the lattice volume by lattice strain and A‐site cation size. It is worth noting that all the materials studied here did not undergo any phase change/distortion under the strains and electric fields considered in this study.

**Figure 2:**
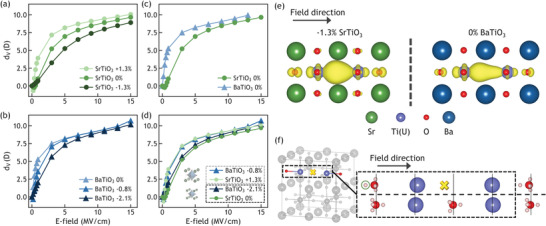
Oxygen vacancy dipole moment *d_V_* in SrTiO_3_ and BaTiO_3_ with field applied parallel to the $Ti^{4+}-V_{O}^{x}-Ti^{4+}$ chain. The *d_V_* for hydrostatically strained a) SrTiO_3_ and b) BaTiO_3_. Comparison of *d_V_* for c) unstrained and d) strained SrTiO_3_ and BaTiO_3_. Legend in (d) groups the SrTiO_3_ and BaTiO_3_ configurations that have the same lattice volume due to applied hydrostatic strains, where + indicates applied hydrostatic tension, and − indicates hydrostatic compression. e) Charge density of two trapped electrons in the vacant site of −1.3% compressed SrTiO_3_ (left) and unstrained BaTiO_3_ (right) under 3 MV cm^−1^. Isosurfaces are taken at 3% of the maximum value, with the $V_{o}^{x}\;$defect energy levels being 0.49 and 0.35 eV below conduction band minimum for SrTiO_3_ and BaTiO_3_, respectively. f) The $O-Ti^{4+}-V_{O}^{x}-Ti^{4+}$ chain in SrTiO_3_, with enlarged view showing the displacement of the sites in the chain (top), comparing with the corresponding sites in defect‐free cell (bottom) at 3 MV cm^−1^. Solid vertical lines represent the origins of each site under zero field, the yellow cross and light pink spheres represent the Wannier centers of the electron pairs of the vacancy trapped electrons and oxide ions, respectively. The Wannier centers on Ti ions are not shown.

In our simulations, we have applied hydrostatic strains to SrTiO_3_ to isolate the effect of lattice volume on polarizability without altering crystal symmetry. **Figure** **2**a shows that the *d_V_* for SrTiO_3_ increased with increasing tensile strain (+1.3% hydrostatic expansion of lattice parameter), relative to unstrained SrTiO_3_ under the same applied field. Hydrostatically compressed (−1.3%) SrTiO_3_ exhibited the lowest *d_V_*, except under the lowest applied field (<0.5 MV cm^−1^). To further validate the role of lattice volume in determining *d_V_*, we altered the A‐site cation from Sr to Ba to increase the lattice volume. At zero strain, BaTiO_3_ exhibited higher *d_V_* than SrTiO_3_, as shown in Figure 2b,c; this is consistent with the expected lattice volume effect. Nevertheless, Ba ions not only result in a larger lattice volume, but alter the A‐site composition. To confirm that the difference between these two compounds arises predominantly from the lattice volume, we strained BaTiO_3_ by −2.1% to match the lattice parameter of unstrained SrTiO_3_, and by −0.8% to match the lattice parameter of +1.3% strained SrTiO_3_. The magnitude of *d_V_* for SrTiO_3_ and BaTiO_3_ were the same in both of these cases with identical lattice volumes, as shown in Figure 2d. The slight differences between unstrained SrTiO_3_ and compressed BaTiO_3_ of the same lattice volume are attributable to the large compressive strain on BaTiO_3_ (Section S5, Supporting Information). These results indicate that the *d_V_* is mainly sensitive to the lattice volume and the effective volume of the oxygen vacancy site when the alkaline earth cations on the A‐site have very similar chemistry (Sr, Ba). Figure 2e also shows the charge density of the two trapped electrons in the vacant site for the smallest and the largest lattice parameters involved in this comparison—the compressed (−1.3%) SrTiO_3_ and the unstrained BaTiO_3_ under 3 MV cm^−1^. The charge distribution in the unstrained BaTiO_3_ is clearly more polarized owing to the larger vacant site space, resulting in higher polarization under the same field strength, compared with that in the compressed (−1.3%) SrTiO_3_.

### 2.3 Site Decomposed Polarization at and around the Oxygen Vacancy

To further understand the underlying physics of *d_V_*, we utilized Wannier calculations^[^ ^39^, ^40^ ^]^ and decomposed *d_V_* into the field‐induced dipole moment of each site in the system. We found that not only the trapped electrons but the whole $O-Ti^{4+}-V_{O}^{x}-Ti^{4+}$ chain promotes *d_V_* via both ionic and electronic displacements, as illustrated in Figure 2f. First, as the Ti cations and trapped electrons are of opposite charge, they were polarized in opposite directions by the electric field: one of the Ti ions in the $Ti^{4+}-V_{O}^{x}-Ti^{4+}$ chain approached the trapped electrons with increasing field, whereas the other ion receded further away. Second, and most importantly, the approaching Ti ion and the Wannier center of the trapped electrons both showed larger displacements than the corresponding sites in the defect‐free cell (Ti and O ion, respectively), contributing strongly and positively to *d_V_*, as shown in Figure 2f. We believe this is a result of the strong attraction between the Ti ion and the two electrons without the confinement of the oxygen atom potential, as well as the ease of reducing the Ti^4 +^  to Ti^3 +^ (high electronegativity of Ti). Third, the O ion in the $O-Ti^{4+}-V_{O}^{x}-Ti^{4+}$ chain also promoted *d_V_* owing to the circled Wannier center with large displacement under electric field shown in Figure 2f. Last, the receding Ti ion only showed slightly larger displacement in the defected cell than the corresponding site in the defect‐free cell. We found that up to 75% of *d_V_* is contributed by the $O-Ti^{4+}-V_{O}^{x}-Ti^{4+}$ defect cluster, whereas other sites further away from the vacant site had polarization comparable to the corresponding sites in the perfect cell (See Section S5, Supporting Information, for detailed site‐decomposed polarization analyses^[^ ^42^ ^]^).

### 2.4 B site Cation Reducibility Effect on Oxygen Vacancy Dipole Moment,  *d_V_*

**Figure 3:**
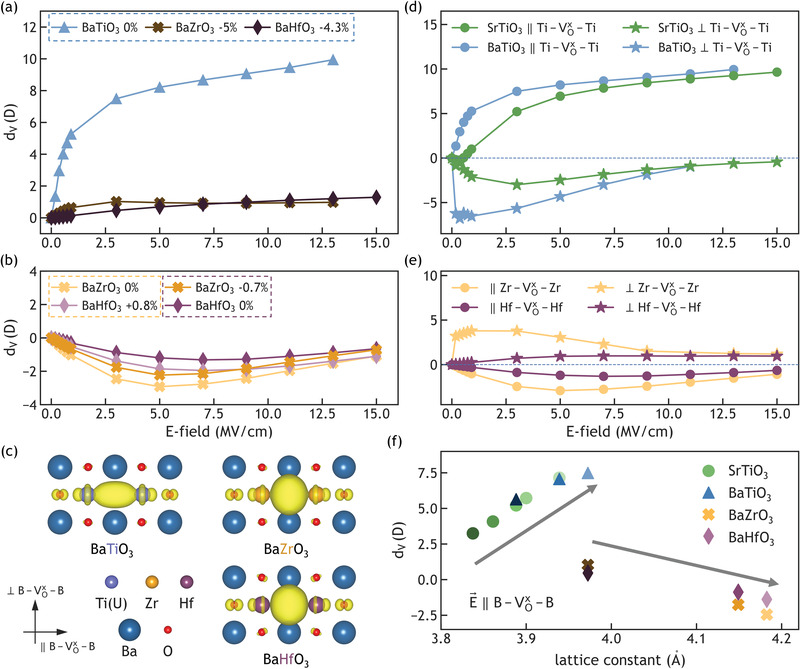
Oxygen vacancy dipole moment *d_V_* and charge density distribution of the trapped electrons in Ba(Ti, Zr, Hf)O_3_. The *d_V_* of BaTiO_3_, BaZrO_3_, and BaHfO_3_ with field applied parallel to $B^{4+}-V_{O}^{x}-B^{4+}$ (*B* = Ti, Zr, Hf), with the latter two compounds a) compressively strained to match the lattice constant of unstrained BaTiO_3_, and b) strained either in tension (BaHfO_3_) or compression (BaZrO_3_) to match the lattice constant of each other. The dotted boxes group the Ba‐based perovskites with the same lattice volume. c) Charge density of the two trapped electrons in BaTiO_3_, BaZrO_3_, and BaHfO_3_ under zero strain and zero field strength, showing distinct isosurface shapes with different B‐site cations. Isosurfaces are taken as 3% of the maximum value for BaTiO_3_, and 10% for BaZrO_3_ and BaHfO_3_ with defect energy levels being 0.35, 0.68, 1.15 eV below conduction band minimum for BaTiO_3_, BaZrO_3_, and BaHfO_3_, respectively. *d_V_* for unstrained d) SrTiO_3_ (green), BaTiO_3_ (blue), and e) BaZrO_3_ (orange), BaHfO_3_ (purple) with field applied parallel (circles) or perpendicular (stars) to the $B^{4+}-V_{O}^{x}-B^{4+}$ chain. f) *d_V_* for all compounds under different strain states and an electric field of 3 MV cm^−1^ parallel to the $B^{4+}-V_{O}^{x}-B^{4+}$ chain as a function of lattice constant of the compound.

Next, to test the hypothesis that the reducibility of the cation affects the polarizability of the trapped electrons nearby, we have used different transition metals on the B‐site of the perovskite, in particular BaTiO_3_, BaZrO_3_, and BaHfO_3_. As we noted above, we use electronegativity of a metal as a simpler measure of the reducibility of the metal cation. Prior work has shown that Ti ions in SrTiO_3_ and TiO_2_ can easily form polaronic defects^[^ ^43^, ^44^ ^]^ (Ti^4 +^ + *e* ^−^ → Ti^3 +^), whereas Zr and Hf ions in ZrO_2_ and HfO_2_, respectively, would be energetically disfavored to form polaronic defects.^[^ ^45^, ^46^, ^47^, ^48^ ^]^ Not all of these *AB*O_3_ compounds (Sr,Ba)(Ti,Hf,Zr)O_3_ are stable in the cubic phase^[^ ^23^, ^49^, ^50^ ^]^ and we observed significant structural distortion in SrZrO_3_ and SrHfO_3_ simply by introducing the oxygen vacancy. Therefore, we focused on the Ba‐based cubic perovskites with different B‐site cations of 4+ valence: Ba*B*O_3_ (*B* = Ti, Zr, Hf), with the electronegativity of Ti, Zr, and Hf being 1.54, 1.33, and 1.30,^[^ ^51^ ^]^ respectively. To facilitate comparison of *d_V_* as a function of electronegativity, we maintained the same lattice constant by imposing different hydrostatic strains to each of the three perovskites. We applied electric field parallel to the $B^{4+}-V_{O}^{x}-B^{4+}$ (*B* = Ti, Zr, Hf) chain and strained BaZrO_3_ and BaHfO_3_ by −5% and −4.3%, respectively, to match the lattice constant of unstrained BaTiO_3_. As shown in **Figure** **3**a, hydrostatically compressed BaZrO_3_ (−5%) and BaHfO_3_ (−4.3%) exhibited similar *d_V_*, and these *d_V_* were much lower than that of unstrained BaTiO_3_. This result confirms that *d_V_* increases with the electronegativity of the B‐site cation neighboring the trapped electrons at the oxygen vacancy site.

However, the simulated compressive strains were impractically large for BaZrO_3_ and BaHfO_3_ (−5% and −4.3%, respectively). To avoid potential artifacts introduced by such large strains, we have also applied smaller strains on BaHfO_3_ (+0.8%) and BaZrO_3_ (−0.7%) to match the lattice constants of unstrained BaZrO_3_ and BaHfO_3_, respectively. First, as shown in Figure 3b, although all four *d_V_* responses were similar in overall trend and magnitude, none were identical even when BaZrO_3_ and BaHfO_3_ had matching lattice volumes. Furthermore, all *d_V_* were negative in both BaZrO_3_ and BaHfO_3_ under zero or small strain when the field was applied parallel to the $B^{4+}-V_{O}^{x}-B^{4+}$ (*B* = Zr, Hf) chain, indicating that this polarization direction is unfavorable in defective BaZrO_3_ and BaHfO_3_, in contrast to SrTiO_3_ and BaTiO_3_. We found a correlation between the favored polarization direction (positive *d_V_*) and the charge density distribution of the trapped electrons in Ba*B*O_3_ (*B* = Ti, Zr, Hf) under zero field and strain. As shown in Figure 3c, the electron charge density is elongated toward to the two neighboring Ti ions in unstrained BaTiO_3_ at zero field. We believe that such a highly anisotropic charge distribution indicates the favored and unfavored polarization directions, which were parallel and perpendicular to the $Ti^{4+}-V_{O}^{x}-Ti^{4+}$ chain, respectively. By contrast, the charge density distribution of $V_{o}^{x\;}$in BaZrO_3_ and BaHfO_3_ is more isotropic, and showed slight repulsion from the neighboring Zr or Hf ions. The resulting slightly elongated charge distribution perpendicular to the $B^{4+}-V_{O}^{x}-B^{4+}$ (*B* = Zr, Hf) chain suggested that the trapped electrons can more favorably polarize perpendicular to the $B^{4+}-V_{O}^{x}-B^{4+}$ (*B* = Zr, Hf) chain, albeit with a weaker polarization.

### 2.5 Favored Polarization Direction

Figure 3d,e shows the *d_V_* for unstrained SrTiO_3_, BaTiO_3_, BaZrO_3_, and BaHfO_3_ with field applied either parallel (circles) or perpendicular (stars) to the $B^{4+}-V_{O}^{x}-B^{4+}$ (*B* = Ti, Zr, Hf) chains. Here, *d_V_* was positive in (Sr, Ba)TiO_3_ when electric field was applied parallel to the $Ti^{4+}-V_{O}^{x}-Ti^{4+}$ chain (the favorable polarization direction). In contrast, BaZrO_3_ and BaHfO_3_ exhibited positive *d_V_* when the field was perpendicular to the $B^{4+}-V_{O}^{x}-B^{4+}$ (*B* = Zr, Hf) chain and negative *d_V_* when the field was parallel to this chain. This confirms our above proposed correlation between the charge distribution shape at zero‐field and the favored polarization direction (sign of *d_V_*). Furthermore, Figure 3d,e also show that increased lattice volume (*V*_SrTiO3_ < *V*_BaTiO3_, *V*_BaHfO3_ < *V*_BaZrO3_) correlated with increasingly positive *d_V_* when field was applied along the favored polarization direction, and more negative *d_V_* when the field was applied perpendicular to the favored polarization direction of the material. In Figure 3f, we summarize *d_V_* with respect to lattice constant under an applied field of 3 MV cm^−1^ (obtained for the four different perovskite compositions with different strains); field was applied parallel to the $B^{4+}-V_{O}^{x}-B^{4+}$ (*B* = Ti, Zr, Hf) chains. Because this is the favored polarization direction in (Sr, Ba)TiO_3_, *d_V_* increased positively and also increased linearly with increasing lattice constant. In contrast, *d_V_* became more negative with increasing lattice volume in Ba(Zr, Hf)O_3_ for which the favored polarization is perpendicular to the $B^{4+}-V_{O}^{x}-B^{4+}$ chain.

The oxygen vacancy dipole moment *d_V_* depends chiefly on the dipole moment of the defect cluster ($O-B^{4+}-V_{O}^{x}-B^{4+}$, *B* = Ti, Zr, Hf) in these *AB*O_3_ perovskite compounds. The dipole moment of the main segment ($B^{4+}-V_{O}^{x}-B^{4+}$) of this defect cluster is determined by the localization of the trapped electrons at $V_{O}^{x}$and their interaction with the neighboring B‐site cation under electric field, both correlated closely with the electronegativity of the B‐site cation.

**Figure 4:**
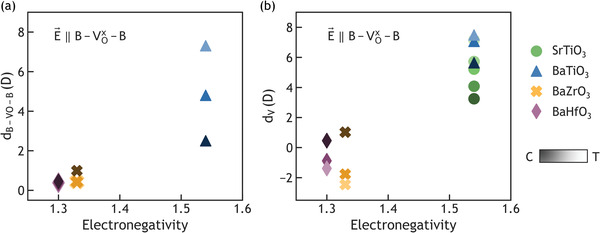
Defect cluster dipole and oxygen vacancy dipole moment with electric field applied parallel to $B^{4+}-V_{O}^{x}-B^{4+}$. a) The defect cluster $B^{4+}-V_{O}^{x}-B^{4+}$ dipole moment, *d*_*B* − *VO* − *B*_, under 1 MV cm^−1^ as a function of electronegativity of the B‐site element. b) The net dipole moment introduced by oxygen vacancy, *d_V_*, for all compounds under different strain states and an electric field of 3 MV cm^−1^ as a function of electronegativity of the B‐site element. Multiple points for each compound represent differently strained lattice parameter, where transitions from darker to lighter shades of a given colored symbol indicate strain variation from relative compression (*C*) to tension (*T*).

**Figure** **4** illustrates magnitudes and trends for the dipole moment of the defect cluster and oxygen vacancy, for several *AB*O_3_ compositions each under varying magnitudes of relative compression or tension. It is worth noting that *d*_*B* − *VO* − *B*_ is a local dipole calculated in the defected crystal, whereas *d_V_* is a difference in the overall dipole moments of both defected and perfect crystals. In Figure 4a, we show the local dipole moment of the $B^{4+}-V_{O}^{x}-B^{4+}$ chain (*B* = Ti, Zr, Hf), *d*_*B* − *VO* − *B*_, with a strong positive correlation to the electronegativity of the B‐site cation. The effect of lattice strain also highly depends on the electronegativity of the B‐site cation, where the strain alters *d*_*B* − *VO* − *B*_ significantly in BaTiO_3_, has a moderate effect on BaZrO_3_, and has minimal effect on BaHfO_3_. Although Zr and Hf are physically and chemically similar, they still have slightly different electronegativities^[^ ^51^ ^]^ and reducibilities,^[^ ^52^ ^]^ resulting in different *d*_*B* − *VO* − *B*_ and *d_V_* under the same lattice volume, as well as in their responses to lattice strain. Finally, Figure 4b illustrates the same data as those in Figure 3f, now expressed as a function of B‐site cation electronegativity in each compound (Ti, Zr, or Hf). Note the strong positive correlation between B‐site cation electronegativity and *d_V_* magnitude, consistent with our hypothesis that B‐site cation reducibility modulates the core defect cluster dipole and the effective *d_V_* under applied field.

In addition to the correlation we have identified between the defect polarization and the electronegativity of the B site cation, we also found that the electron effective mass at the conduction band minimum (CBM) correlated with defect polarization. We believe that the more reducible the B site cation (equivalent to higher electronegativity), the more easily it can “trap” the electron, which will lead to larger electron effective mass. Our band structure calculations on BaBO_3_ (*B* = Ti, Zr, Hf) with the same lattice constant showed that both *d*_*B* − *VO* − *B*_ and *d_V_* correlate positively with the electron effective masses at the conduction band minimum (CBM), similar to the correlation shown in Figure 4 (Section S6, Supporting Information).

## 3 Conclusions

In summary, a direct relationship exists between lattice volume and oxygen vacancy dipole moment in *AB*O_3_ perovskites as a function the A‐site cation size and/or lattice strain. This correlation affects the response of such materials to applied electric field. Expanding the lattice volume increases the net dipole of the defective system *d_V_*, if the field is applied in the direction of favorable polarization. In fact, with the same B‐site cation, SrTiO_3_ and BaTiO_3_ strained to exhibit the same lattice constants and also exhibited identical *d_V_*. The B‐site cation species independently played an important role in affecting *d_V_*, as illustrated for Ba(Ti, Zr, Hf)O_3_ in which the B site cation electronegativity correlated positively with *d_V_* and the local dipole moment of the defect cluster *d*_*B* − *VO* − *B*_. The electronegativities of the B site cations thus showed great influence on defect formation energies under electric field. The preferred polarization direction of defects also varied with the effective electronegativity of the B‐site cation, which indicated different defect ordering, parallel or perpendicular, with respect to the field. The local Hubbard *U* approach presented in this work provides a cost‐efficient and presently accessible method to study defected semiconducting perovskite oxides under electric field, while having some limitations regarding the electronic structure of the material. We believe our framework provides a general method for the analysis of defected semiconducting oxides under electric field. Our findings regarding the stability and properties of defects that are polarized under strong electric fields can enable development of more accurate physical models of predicting the behavior of thin‐film redox based memristors.^[^ ^1^, ^2^ ^]^

## 4 Methods

In this study, the authors conducted all DFT and Berry phase simulations^[^ ^17^, ^18^ ^]^ with the Quantum Espresso package version 6.4.1.^[^ ^21^, ^22^ ^]^ The plane‐wave kinetic energy cutoff was set to 100 Ry and charge density cutoff to 400 Ry. Optimized norm‐conserving Vanderbilt pseudopotentials, NCSR (ONCVPSP v0.4), from PseudoDojo^[^ ^53^, ^54^ ^]^ with Perdew–Burke–Ernzerhof exchange‐correlation functional for solids (PBEsol)^[^ ^55^ ^]^ are used in this study. Lattice constants for *AB*O_3_ (*A* = Ca, Sr and Ba; *B* = Ti, Zr and Hf) were obtained using 21 different volumes, fitted with the 3rd order Birch–Murnaghan equation of state.^[^ ^56^ ^]^ Reciprocal space was sampled using 8 × 8 × 8 displaced Monkhorst‐Pack k‐point grid^[^ ^57^ ^]^ with no smearing in all calculations. All compounds were fitted under cubic symmetry constraint.

The authors conducted electric field simulations on SrTiO_3_, BaTiO_3_, BaZrO_3_, and BaHfO_3_ with Berry phase approach (lelfield = .true.) and modern theory of polarization.^[^ ^17^, ^58^ ^]^ Local Hubbard *U* correction was applied on Ti but not on Zr nor Hf, since wide bandgap materials such as BaZrO_3_ and BaHfO_3_ do not require such a correction to obtain a correct in‐gap defect state under electric field. Hubbard *U* was applied using the DFT + *U* simplified version by Cococcioni and de Gironcoli,^[^ ^34^ ^]^ which corresponded to “lda_plus_u_kind = 0” in Quantum ESPRESSO. The authors used 2 × 2 × 2 supercell on both perfect and defective systems, with reciprocal space sampled using 2 × 2 × 2 Monkhorst‐Pack k‐point grid,^[^ ^57^ ^]^ displaced by (0.5 0.5 0.5).

For field applied either parallel or perpendicular to the $B^{4+}-V_{O}^{x}-B^{4+}\;$(*B* = Ti, Zr, Hf) chain, the authors relaxed the system fully within two field strength regimes: low field {0, 0.00005, 0.00010, 0.00015, 0.00020, 0.00025} in Ry atomic units, and high field {3, 5, 7, 9, 11, 13, 15} MV cm^−1^. Note that 0.00005 Ry is ≈0.2 MV cm^−1^. Under every field, electronic and ionic structures were allowed to fully relax, while the shape and lattice constant were constrained. They set the convergence condition for electronic structure relaxation to be 10^−9^ Ry, and ionic relaxation stopping criteria to be 4 × 10^−6^ Ry for total energy and 4 × 10^−5^ Ry/bohr for forces. Spin was not considered in their calculations. They adopted their previous method^[^ ^5^ ^]^ and applied small Gaussian smearing (0.004 Ry) to speed their calculation with no fractional occupations

## Conflict of Interest

The authors declare no conflict of interest.

## Supporting information

Supporting Information

## Data Availability

The data that support the findings of this study are available from the corresponding author upon reasonable request.
