# Supplementary material for: Complex Oxides under Simulated Electric Field: Determinants of Defect Polarization in ABO3 Perovskites
Source: Adv Sci (Weinh). 2021 Dec 10;9(4):2104476. doi: 10.1002/advs.202104476 (PMC8811848; doi:10.1002/advs.202104476)
Supplement: Supplementary file 1 — Supporting Information [file ADVS-9-2104476-s001.pdf]

© 2021 Wiley-VCH GmbH

Supporting Information

for *Adv. Sci.,* DOI: 10.1002/advs.202104476

Complex oxides under simulated electric field: Determinants of defect polarization in ABO_3 perovskites

Yen-Ting Chi[1], *Krystyn J. Van Vliet*[1*], *Mostafa Youssef*[1,2*] *and Bilge Yildiz*[1,3*]

Supplemental materials for Complex oxides under simulated electric field: Determinants of defect polarization in ABO3 perovskites

Yen-Ting Chi[1], Krystyn J. Van Vliet[1*], Mostafa Youssef[1,2*] and Bilge Yildiz[1,3*]

[1]*Department of Materials Science & Engineering, Massachusetts Institute of Technology, Cambridge, Massachusetts 02139 USA*

[2]*Department of Mechanical Engineering, The American University in Cairo, AUC Avenue, P.O. Box 74, New Cairo 11835 Egypt*

[3]*Department of Nuclear Science & Engineering, Massachusetts Institute of Technology, Cambridge, Massachusetts 02139 USA*

Email: krystyn@mit.edu; mostafa.youssef@aucegypt.edu; byildiz@mit.edu

List of content

1. **Computational details**
    a) **Density functional theory and material property details**
    b) **Electric field calculation – Berry phase details**
2. **Local Hubbard U method**
    a) **Hubbard U values comparison**
    b) **Role of d_V in defect formation energy**
3. **Strain effect on dielectric and electronic properties of STO**
4. **Electric field direction perpendicular to $Ti^{4+} - V_o^x - Ti^{4+}$ chain – octahedral rotation**
5. **Wannier calculation details and site-decomposed polarization**
    a) **Site-decomposed polarization – STO and BHO**
    b) **Compressive strain effect on negative d_V under low field**
    c) **Favored polarization direction and strain effect on d_V**
    d) **Compressive strain promotes d_V in BHO and BZO**
6. **Electronegativity, d_V and d_{B-VO-B}**

Supplemental References

1. Computational details

a) Density functional theory and material property details

In this study, we conducted all density functional theory and Berry phase simulations[1,2] with the Quantum Espresso package version 6.4.1[3,4]. The plane-wave kinetic energy cutoff was set to 100 Ry and charge density cutoff to 400 Ry. Optimized norm-conserving Vanderbilt pseudopotentials, NCSR (ONCVPSP v0.4), from PseudoDojo[5,6] with Perdew-Burke-Ernzerhof exchange-correlation functional for solids (PBEsol)[7] are used in this study. Lattice constants for ABO_3 (A = Ca, Sr and Ba; B = Ti, Zr and Hf) were obtained using 21 different volumes, fitted with the 3rd order Birch-Murnaghan equation of state[8]. Reciprocal space was sampled using 8x8x8 displaced Monkhorst-Pack k-point grid[9] with no smearing in all calculations. All compounds were fitted under cubic symmetry constraint. Fitted parameters for all compounds are shown in Figure S1.

With the fitted lattice constants combined with our local Hubbard U method (details in Section 2) on $ATiO_3$ (A = Ca, Sr and Ba), we constructed 2x2x2 supercells for defect calculations. However, $CaZrO_3$, $CaHfO_3$ and $SrZrO_3$ showed distorted structure by simply having an oxygen vacancy present without applying electric field; $CaTiO_3$ and $SrHfO_3$ showed distorted structure after applying very low field strength parallel to the $B^{4+} - V_o^x - B^{4+}$ chain (B = Ti and Hf). Since the cubic phase is the high temperature phase for these compounds[10–12], the cubic symmetry constraint on these materials is unreasonable and made the supercells very unstable. By introducing an oxygen vacancy, the ions obtained more degrees of freedom, resulting in the distorted structures shown in Figure S2. In this work, we would want to conduct our simulations on materials under the same crystallographic symmetry. Therefore, we only selected $SrTiO_3$ (STO), $BaTiO_3$ (BTO), $BaZrO_3$ (BZO), and $BzHfO_3$ (BHO) in cubic phase to study their defect polarization responses under different electric field and strain. It is worth noting that we did not purposely enforce high structural symmetry to our materials except for $BaTiO_3$. The structural ground state for $BaZrO_3$, $BzHfO_3$ and $SrTiO_3$ are already cubic phase under room temperature[10,13], and selecting cubic phase $BaTiO_3$ is necessary to provide reasonable comparison among cubic structures. We have shown the summary of the equilibrium lattice

constant, bulk modulus, and band gap for the selected cubic phase perovskites in Table S1, along with the selected experimental values for each property. To ensure that the supercell size is big enough with minimal effect on our results, we have conducted additional simulation using a 3x3x3 $SrTiO_3$ supercell. As shown in Figure S3, both the relative electric Gibbs free energy of formation for oxygen vacancy and oxygen vacancy dipole moment in unstrained $SrTiO_3$ showed similar values and trend in different supercell sizes (2x2x2 vs. 3x3x3), indicating that 2x2x2 supercell is big enough for our calculation.

Previous work[14,15] showed that there are four possible configurations of neutral oxygen vacancy in $SrTiO_3$. In our work, with spin polarized calculation and different magnetic moment initializations, we found that nonmagnetic state (singlet state) was the most stable configuration for such defect. Even with non-zero starting magnetic moment on the Ti ions adjacent to the defect site, the system would eventually relax to zero magnetic moment state. If we enforced magnetic solution for the defect (triplet state), the energy of ferromagnetic state was higher than the nonmagnetic state under all field strength as shown in Figure S4. Therefore, we focused on the ground state of neutral oxygen vacancy, singlet state with zero magnetic moment, in our current work. Amore recent study has used hybrid functionals[16] and showed that both electrons of neutral oxygen vacancy should be trapped in the in-gap defect state with a zero magnetic moment. With self-consistent DFT+U methods[15] and comprehensive surveys of all defect configurations, Ricca et al. have also shown that ferromagnetic vacancy is not always favored and one had to enforce Ti atoms adjacent to the vacant site at a specific distance to stabilize the ferromagnetic defect

Table S1. Comparison between our DFT (local Hubbard U on STO and BTO) calculated lattice constant, bulk modulus, and band gap of the cubic perovskites considered in this work and selected experimental values.

Oxides	Lattice constant (Å)		Bulk Modulus (GPa)		Band Gap (eV)	
	This work	Experiment	This work	Experiment	This work	Experiment
STO	3.8882	3.900[13]	186	179[17]	2.46	3.25[18]
BTO	3.9725	4.00[17]	180	162[17]	2.35	3.2[19]
BZO	4.1826	4.1955[10]	163	126[20]*	3.73	4.9[10]
BHO	4.1491	4.1705[10]	172	116[21]*	4.18	5.4[10]

*Polycrystalline samples

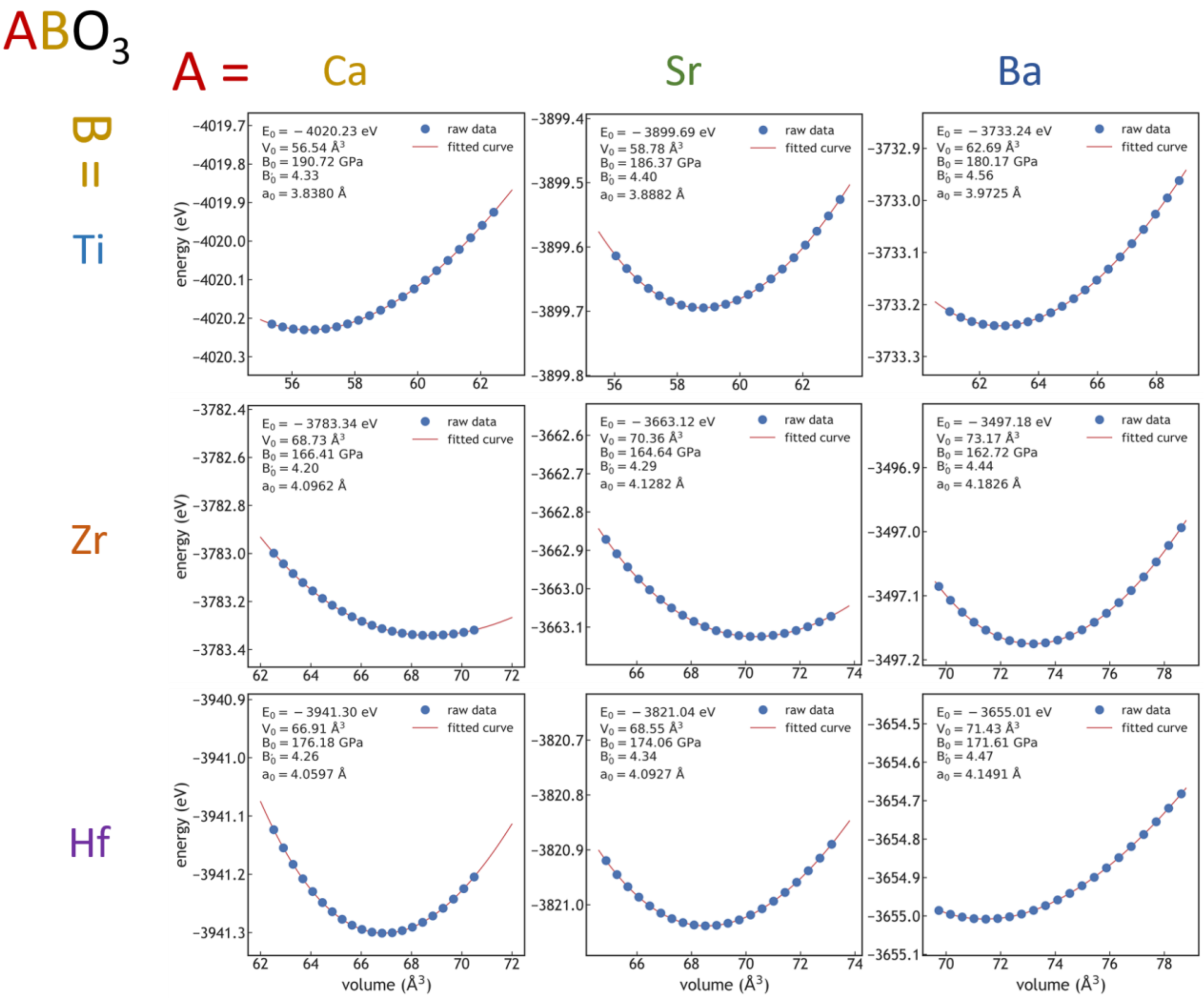

Figure S1. BM fitting for ABO3 (A = Ca, Sr and Ba; B = Ti, Zr and Hf) with 8x8x8 displaced Monkhorst-Pack k-point grid[9]. E_0, V_0, B_0, B_0' and a_0 are the equilibrium energy, equilibrium volume, bulk modulus, first derivative of bulk modulus with respect to volume, and equilibrium lattice constant, respectively.

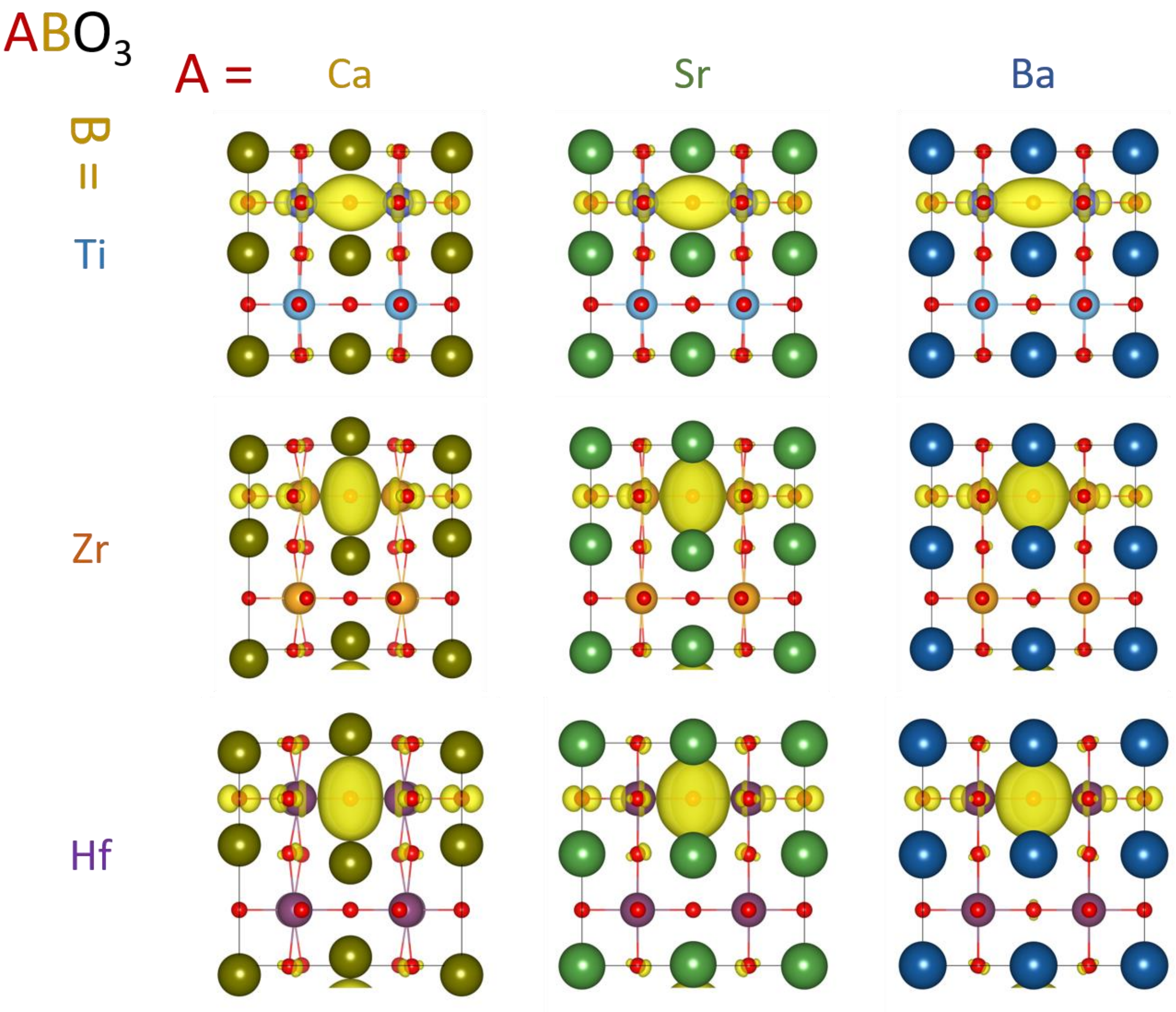

Figure S2. 2x2x2 supercell with one neutral oxygen vacancy for ABO3 (A = Ca, Sr and Ba; B = Ti, Zr and Hf). Yellow isosurfaces represent the charge density distribution in the vacant site.

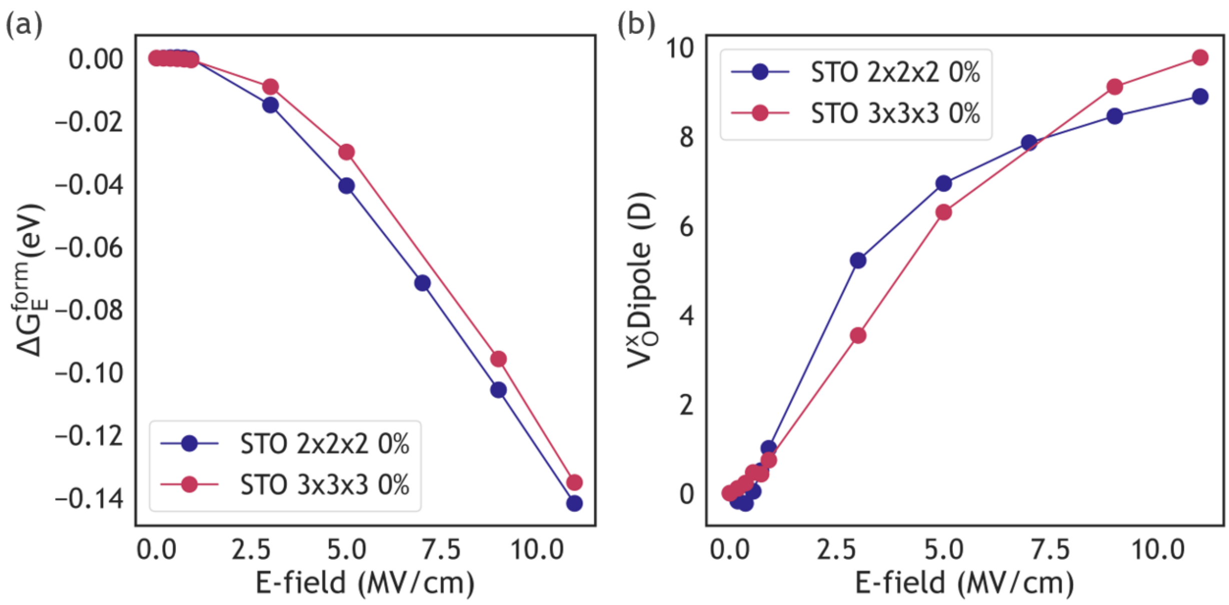

Figure S3. Relative electric Gibbs free energy of formation of oxygen vacancy (a) and oxygen vacancy dipole moment (b) with respect to electric field in 2x2x2 (blue) and 3x3x3 (red) $SrTiO_3$ (STO) supercell under zero strain (0%).

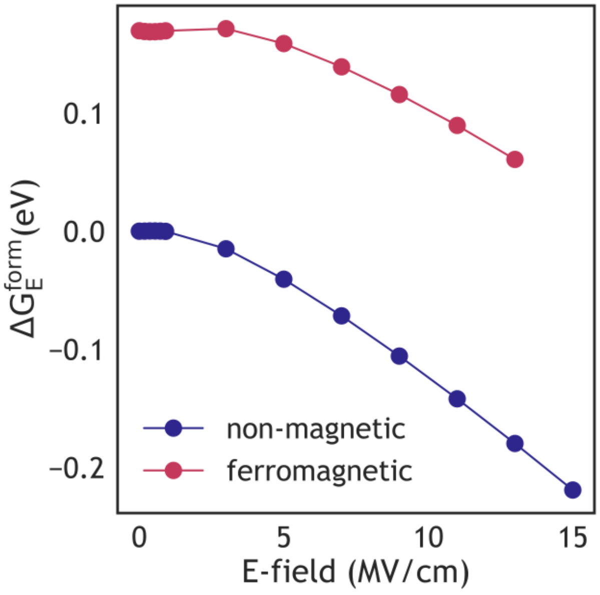

Figure S4. Relative electric Gibbs free energy of formation of oxygen vacancy in $SrTiO_3$ for non-magnetic state (blue) and ferromagnetic state (red). Relative electric Gibbs free energies were plotted using the electric Gibbs free energy of non-magnetic state under zero field strength as reference point (zero).

b) Electric field calculation – Berry phase details

In this study, we conducted electric field simulations on STO, BTO, BZO, and BHO with a local Hubbard U method (for STO and BTO), Berry phase approach (lelfield = .true.) and modern theory of polarization[1,22]. Hubbard U was applied using the DFT+U simplified version by Cococcioni and de Gironcoli[23], which corresponded to "lda_plus_u_kind = 0" in Quantum ESPRESSO. We used 2x2x2 supercell on both perfect and defective systems, with reciprocal space sampled using 2x2x2 Monkhorst-Pack k-point grid[9], displaced by (0.5 0.5 0.5). For field applied either parallel or perpendicular to the $B^{4+} - V_O^x - B^{4+}$ $(B = Ti, Zr\ and\ Hf)$ chain, we relaxed the system fully within two field strength regimes: low field {0, 0.00005, 0.00010, 0.00015, 0.00020, 0.00025} in Ry atomic units, and high field {3, 5, 7, 9, 11, 13, 15} MV/cm. Note that 0.00005 Ry is approximately 0.2 MV/cm. Under every field, electronic and ionic structures were allowed to fully relax, while the shape and lattice constant were constrained. We set the convergence condition for electronic structure relaxation to be 10^{-9} Ry, and ionic relaxation stopping criteria to be $4x10^{-6}$ Ry for total energy and $4x10^{-5}$ Ry/bohr for forces. We have shown the electronic density of states (DOS) for each compound under all field strengths in Figure S5. We adopted our previous method[24], and applied small Gaussian smearing (0.004 Ry) to speed up our calculation which did not show any fractional occupations. Nevertheless, we tested the polarization behavior for unstrained STO with three different settings – 2x2x2 k-point with smearing, 2x2x2 k-point without smearing, and 2x3x2 k-point without smearing (for Wannier calculations), which all showed identical polarization response under all fields as shown in Figure S6. We have also validated that all materials in our study did not undergo any phase change/distortion under the strain and electric fields considered in this study. We examined it by performing a backward relaxation (relaxing the structure from finite field to zero field), and observed no finite polarization from the structure at zero field. We limited the tensile strain on $SrTiO_3$ to be smaller than 2% (up to +1.3%) and on compressive strain on $BaTiO_3$ to avoid severe phase distortion.

In the main manuscript, we have shown the static permittivity of $SrTiO_3$ under all fields using local Hubbard U method with the lattice constant determined by DFT. Due to the smaller lattice constant obtained by DFT (3.8882 Å) compared to experiment measurement (3.900 Å), the static permittivity was underestimated. In Figure S7, we have shown the static permittivity of $SrTiO_3$

using local Hubbard U method with experimental determined lattice constant (green), which were extremely close to the measured values obtained from previous experimental work[25] (orange) under all fields.

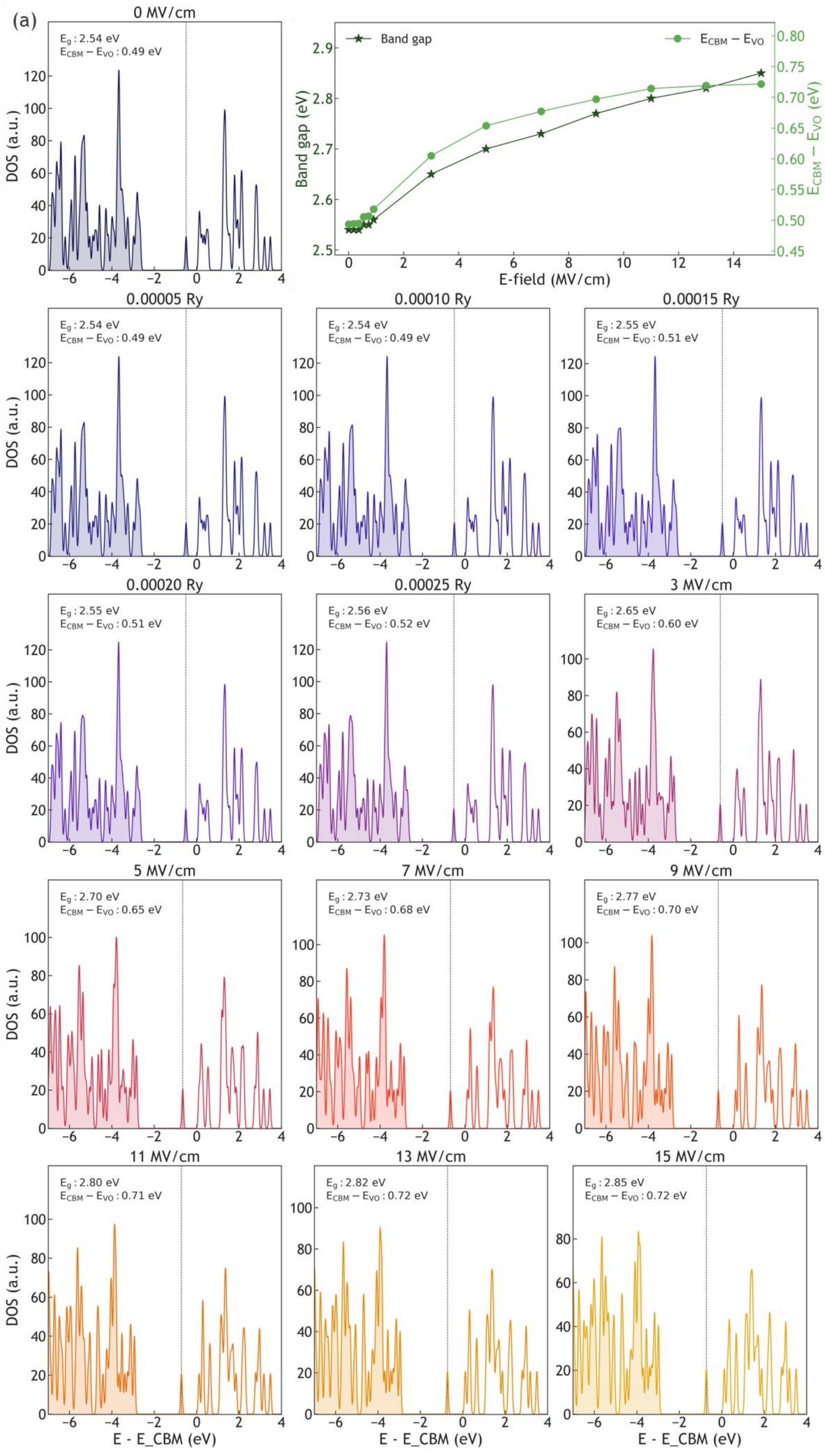
(a)
0 MV/cm
E_g : 2.54 eV
E_CBM − E_VO : 0.49 eV
Band gap
E_CBM − E_VO
Band gap (eV)
E_CBM − E_VO (eV)
E-field (MV/cm)
0.00005 Ry
E_g : 2.54 eV
E_CBM − E_VO : 0.49 eV
0.00010 Ry
E_g : 2.54 eV
E_CBM − E_VO : 0.49 eV
0.00015 Ry
E_g : 2.55 eV
E_CBM − E_VO : 0.51 eV
0.00020 Ry
E_g : 2.55 eV
E_CBM − E_VO : 0.51 eV
0.00025 Ry
E_g : 2.56 eV
E_CBM − E_VO : 0.52 eV
3 MV/cm
E_g : 2.65 eV
E_CBM − E_VO : 0.60 eV
5 MV/cm
E_g : 2.70 eV
E_CBM − E_VO : 0.65 eV
7 MV/cm
E_g : 2.73 eV
E_CBM − E_VO : 0.68 eV
9 MV/cm
E_g : 2.77 eV
E_CBM − E_VO : 0.70 eV
11 MV/cm
E_g : 2.80 eV
E_CBM − E_VO : 0.71 eV
13 MV/cm
E_g : 2.82 eV
E_CBM − E_VO : 0.72 eV
15 MV/cm
E_g : 2.85 eV
E_CBM − E_VO : 0.72 eV
DOS (a.u.)
E - E_CBM (eV)

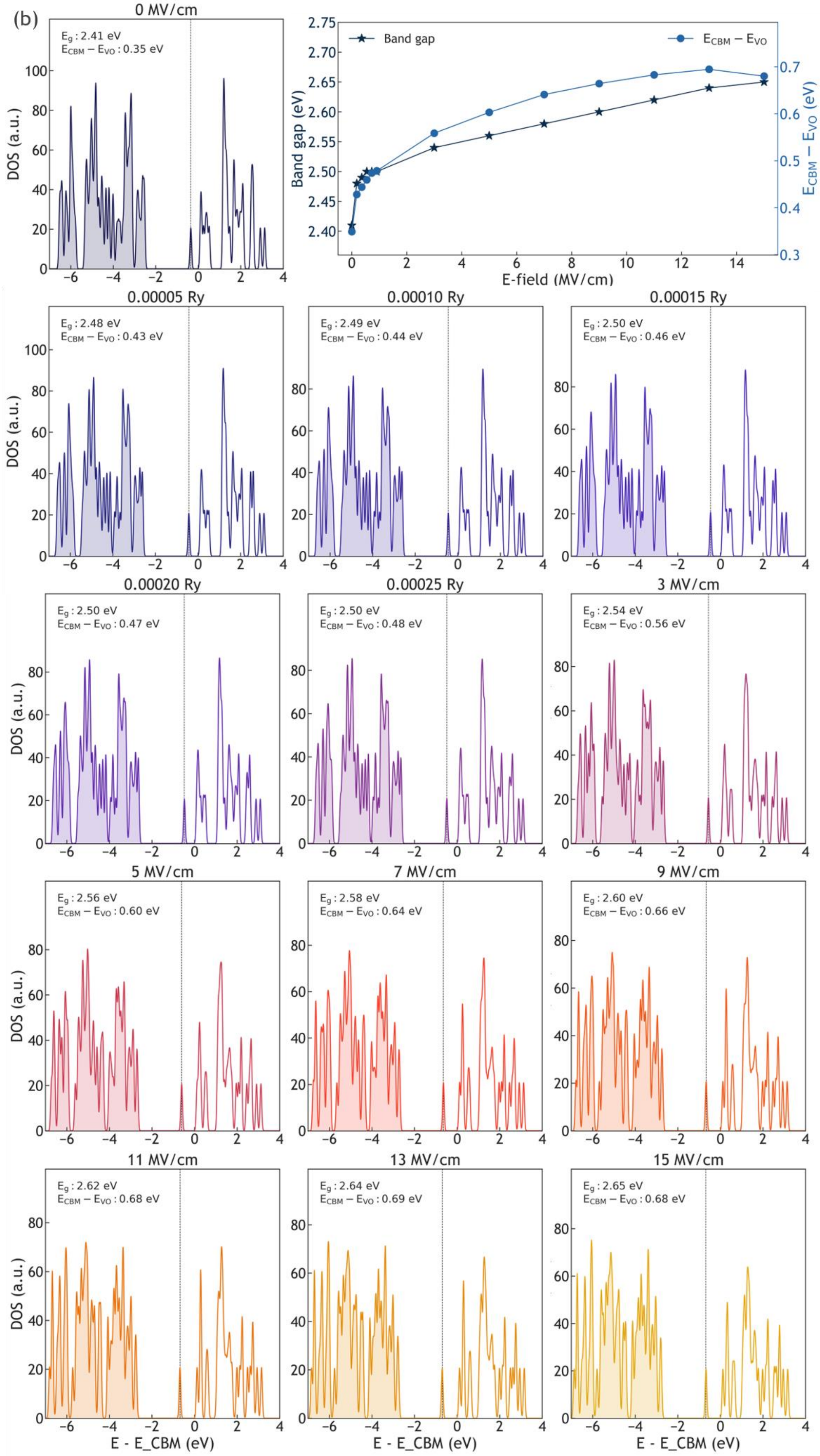
(b)
0 MV/cm
E_g : 2.41 eV
$E_{CBM} - E_{VO}$: 0.35 eV
Band gap
$E_{CBM} - E_{VO}$
Band gap (eV)
$E_{CBM} - E_{VO}$ (eV)
E-field (MV/cm)
0.00005 Ry
E_g : 2.48 eV
$E_{CBM} - E_{VO}$: 0.43 eV
0.00010 Ry
E_g : 2.49 eV
$E_{CBM} - E_{VO}$: 0.44 eV
0.00015 Ry
E_g : 2.50 eV
$E_{CBM} - E_{VO}$: 0.46 eV
0.00020 Ry
E_g : 2.50 eV
$E_{CBM} - E_{VO}$: 0.47 eV
0.00025 Ry
E_g : 2.50 eV
$E_{CBM} - E_{VO}$: 0.48 eV
3 MV/cm
E_g : 2.54 eV
$E_{CBM} - E_{VO}$: 0.56 eV
5 MV/cm
E_g : 2.56 eV
$E_{CBM} - E_{VO}$: 0.60 eV
7 MV/cm
E_g : 2.58 eV
$E_{CBM} - E_{VO}$: 0.64 eV
9 MV/cm
E_g : 2.60 eV
$E_{CBM} - E_{VO}$: 0.66 eV
11 MV/cm
E_g : 2.62 eV
$E_{CBM} - E_{VO}$: 0.68 eV
13 MV/cm
E_g : 2.64 eV
$E_{CBM} - E_{VO}$: 0.69 eV
15 MV/cm
E_g : 2.65 eV
$E_{CBM} - E_{VO}$: 0.68 eV
DOS (a.u.)
E - E_CBM (eV)

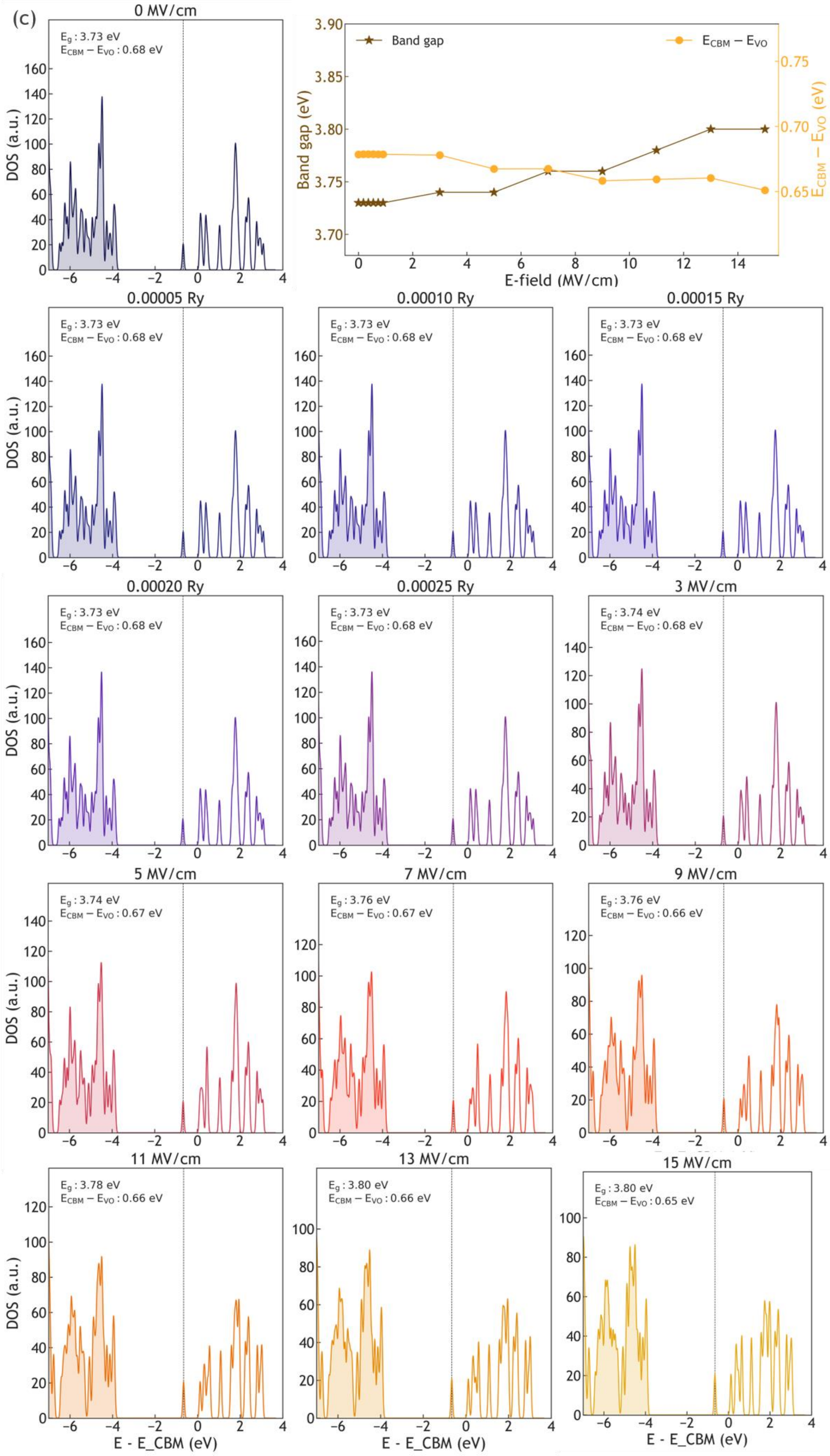

(c)
0 MV/cm
Eg : 3.73 eV
ECBM − EVO : 0.68 eV
DOS (a.u.)
Band gap
ECBM − EVO
Band gap (eV)
ECBM − EVO (eV)
E-field (MV/cm)
0.00005 Ry
Eg : 3.73 eV
ECBM − EVO : 0.68 eV
0.00010 Ry
Eg : 3.73 eV
ECBM − EVO : 0.68 eV
0.00015 Ry
Eg : 3.73 eV
ECBM − EVO : 0.68 eV
0.00020 Ry
Eg : 3.73 eV
ECBM − EVO : 0.68 eV
0.00025 Ry
Eg : 3.73 eV
ECBM − EVO : 0.68 eV
3 MV/cm
Eg : 3.74 eV
ECBM − EVO : 0.68 eV
5 MV/cm
Eg : 3.74 eV
ECBM − EVO : 0.67 eV
7 MV/cm
Eg : 3.76 eV
ECBM − EVO : 0.67 eV
9 MV/cm
Eg : 3.76 eV
ECBM − EVO : 0.66 eV
11 MV/cm
Eg : 3.78 eV
ECBM − EVO : 0.66 eV
13 MV/cm
Eg : 3.80 eV
ECBM − EVO : 0.66 eV
15 MV/cm
Eg : 3.80 eV
ECBM − EVO : 0.65 eV
E - E_CBM (eV)

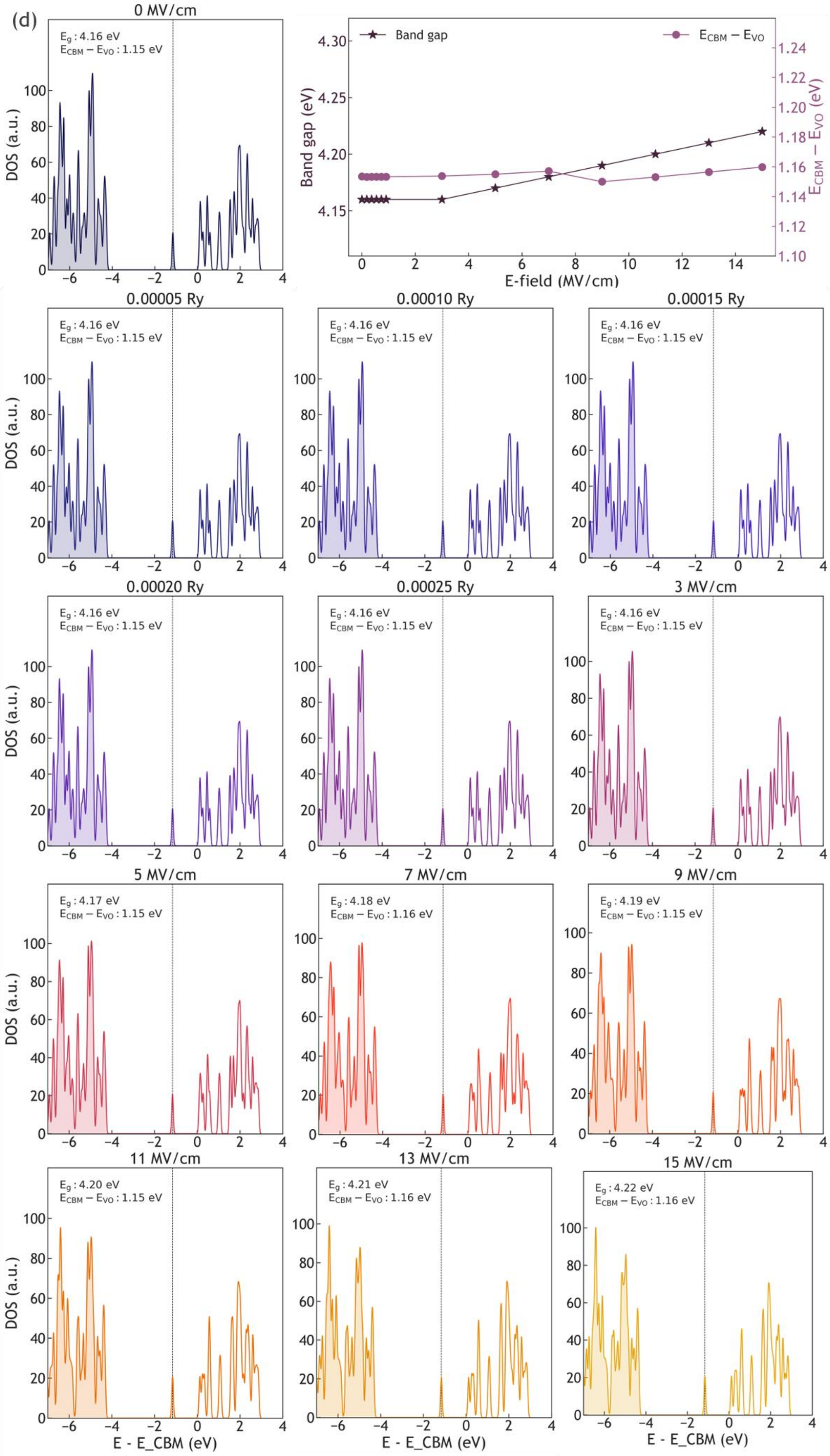

(d)
0 MV/cm
E_g : 4.16 eV
$E_{CBM} - E_{VO}$: 1.15 eV
Band gap
$E_{CBM} - E_{VO}$
Band gap (eV)
$E_{CBM} - E_{VO}$ (eV)
E-field (MV/cm)
0.00005 Ry
0.00010 Ry
0.00015 Ry
0.00020 Ry
0.00025 Ry
3 MV/cm
5 MV/cm
E_g : 4.17 eV
7 MV/cm
E_g : 4.18 eV
$E_{CBM} - E_{VO}$: 1.16 eV
9 MV/cm
E_g : 4.19 eV
11 MV/cm
E_g : 4.20 eV
13 MV/cm
E_g : 4.21 eV
15 MV/cm
E_g : 4.22 eV
DOS (a.u.)
E - E_CBM (eV)

Figure S5. Density of states (DOS) for (a) STO, (b) BTO, (c) BZO, and (d) BHO under all field strengths. DOS are plotted against energies with the bottom of conduction band (E_{CBM}) being the reference point (zero). Dotted lines indicate the position of the defect states in the band gap. Band gaps and the energy difference between the defect state and the bottom of the conduction band ($E_{CBM} - E_{VO}$) are shown at the top left corner in each DOS plot. Band gaps and $E_{CBM} - E_{VO}$ are also plotted with respect to the applied electric field.

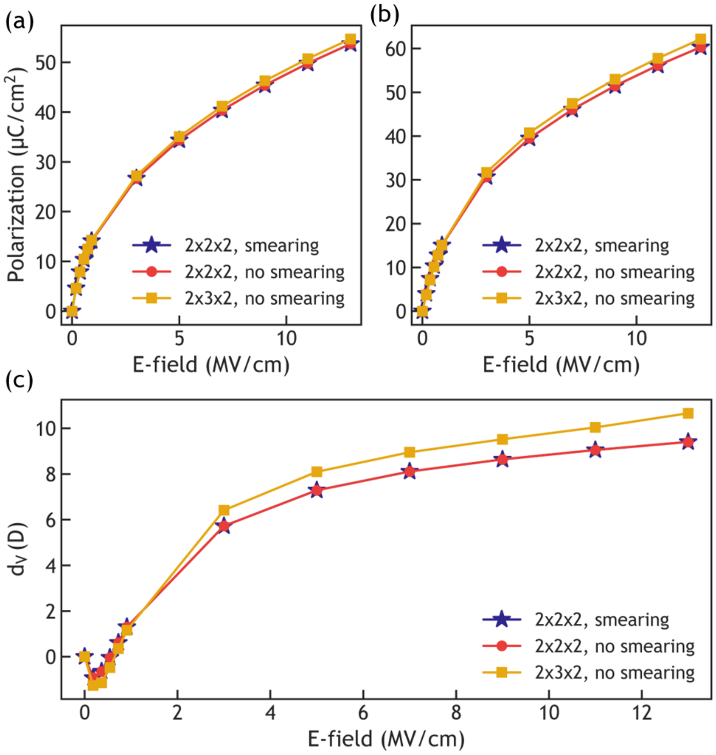

Figure S6. Polarization of (a) perfect and (b) defective STO supercell, and (c) d_V with respect to electric field with different nxnxn K point grids and smearing settings.

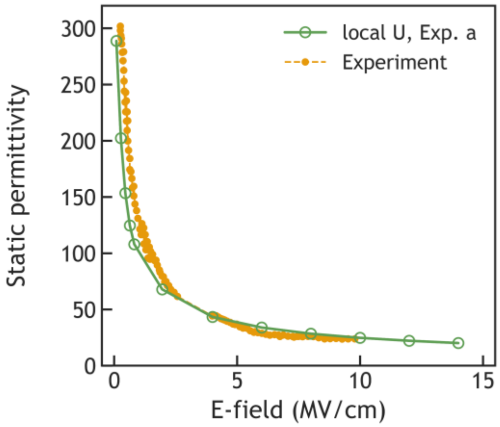

Figure S7. Static permittivity obtained with local Hubbard U method with experimental determined lattice constant (green) and measured values replotted from previous experimental work[25] (orange).

2. Local Hubbard U method

a) Hubbard U values comparison

As discussed in the main text, for STO with oxygen vacancies, Hubbard U is necessary to obtain the correct in-gap defect states; otherwise, a system with vacancies would result in a questionable metallic solution under electric field[26,27]. In Figure S8, we have shown the integrated local DOS of the two electrons which occupied the highest energy states under zero field using traditional DFT method and our local Hubbard U method. It is clear that even without applying electric field, the electrons have spread to the Ti t_{2g} band, forming the $3d_{yz}$ orbital shape. Without Hubbard U on Ti 3d states, STO would form metallic solution under very low field strength. However, previous studies have also shown that the material polarization response under electric field would be greatly underestimated by the Hubbard U[28–30].

In order to resolve this dilemma, we first tried combining Berry phase with hybrid functionals, hoping to achieve correct electronic and ionic structure at the same time. Nevertheless, although there was no theoretical reason that these two approaches should not work together, the current Quantum Espresso package structure was not implemented to use these two methods simultaneously. It appeared that the iteration processes of these two approaches interfere with each other, and would result in questionable results for self-consistent single-shot calculation, and would fail to converge for ionic relaxation[31,32]. Therefore, we returned to the DFT+U based method, and altered it by only introducing Hubbard U on the two Ti ions adjacent to the vacant site.

We first tested the Hubbard U values on Ti for localizing the defect in-gap state with 0.1, 1, 2.5 and 5 eV, where only 5 eV of Hubbard U value was able to provide integer occupation numbers, and lower Hubbard U values showed fractional occupation numbers. We then did a systematic survey on the influence of U values (3eV, 4eV and 5eV) with 2x2x2 k-point gird on different physical properties like polarization, d_V, formation energies, band gaps and $E_{CBM} - E_{VO}$ under different field strengths. As shown in Figure S9, different U values affect the defected system under low fields more obviously. However, the trend of the curves remained consistent, indicating that all our observations and analyses still hold independent of the selected U value. To select the most adequate Hubbard U value in this study, we further tested the defected STO

under electric field with denser k-point grid (from 2x2x2 to 2x3x2). Using Hubbard U values equal to 3eV and 4eV, we obtained metallic solution for defected STO under field strength 9MV/cm and 11MV/cm, respectively, while U value equal to 5eV showed consistent results up to 13MV/cm. Furthermore, recently self-consistent Hubbard U study on STO also suggested the ideal Hubbard U value on the neighboring Ti ions of the neutral oxygen vacancy being 4.8eV[15]. With the equation describing the relationship between breakdown field and band gap for binary high-k dielectrics[33], we could estimate the intrinsic breakdown field for STO being above 11MV/cm (although STO is not a binary oxide). Recent experimental study also showed that the breakdown field for BTO thin film can reach up to 8MV/cm in a heterojunction device[34]. From the estimations above, we believe the intrinsic breakdown field for STO should be above 10MV/cm in our simulation, since there was no geometric factor that could result in electric field concentration or tunneling in the simulation, causing the intrinsic breakdown field strength in simulation being much higher compared to experimental values. Moreover, the previous study also did a systematic survey of Hubbard U determination for transition metal binary oxides[35], where a Hubbard U value equal to 5eV showed the best results on Ti ions. Although the pseudopotentials used in that study, GBRV[36] ultrasoft pseudopotential[37], were different from the pseudopotentials used in our study, we believe it still provided important insight for Hubbard U value determination. Therefore, we selected 5eV to be our local Hubbard U value for STO and BTO in this study. Note that the local Hubbard U was used in both defected and perfect structures on the corresponding Ti ions to be consistent. Using the selected U values and parameters described in previous sections, we obtained band gaps of 2.46 eV for perfect STO, and 2.54 eV for defected STO. The underestimation of band gaps compared to the experimental value (3.2eV[19]) was attributed to the lack of Hubbard U corrections on all the Ti and O ions in. With $\mu_{O_2} = \mu_{O_2}^{ref} + E_{O_2}^{DFT}$, where μ_{O_2} being the oxygen chemical potential, $\mu_{O_2}^{ref}$ being the difference in chemical potential of O_2 between the reference condition and the condition of interest, and $E_{O_2}^{DFT}$ being the total energy of an isolated oxygen molecule computed by DFT, we set $\mu_{O_2}^{ref}$ to be zero to reflect oxygen rich condition, and obtained absolute oxygen vacancy formation energy under zero field of 5.41 eV. This is comparable to 6.2 eV under the same oxygen-rich conditions in ref[16] using HSE hybrid functionals.

For wide bandgap semiconductors (> 3.5eV from density functional theory simulation) such as BZO and BHO, local Hubbard U correction is not required to obtain correct in-gap defect state under facilitating subsequent application of the electric field. Nevertheless, we were curious about how the local U correction could affect the oxygen vacancy dipole moment even when such correction is not needed, especially when comparing to $BaTiO_3$. In Figure S 10, we have shown the oxygen vacancy dipole moment for BHO under compression, and we found that the influence of local Hubbard U correction on such wide bandgap material is very small.

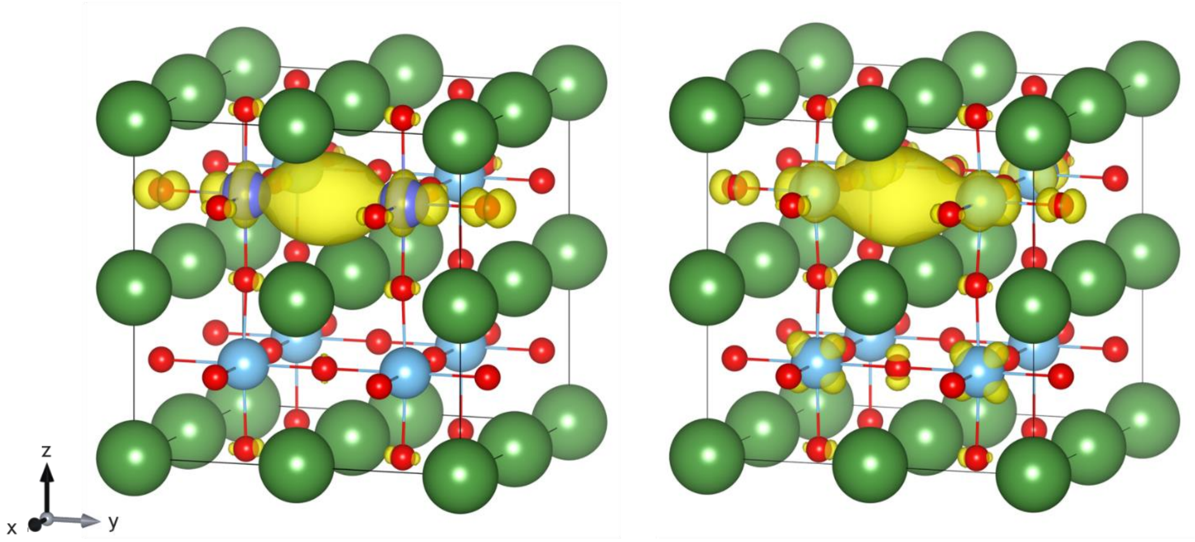

Figure S8. Integrated local density of states of the two electrons occupying the highest energy states with local Hubbard U method (left) and traditional DFT method (right).

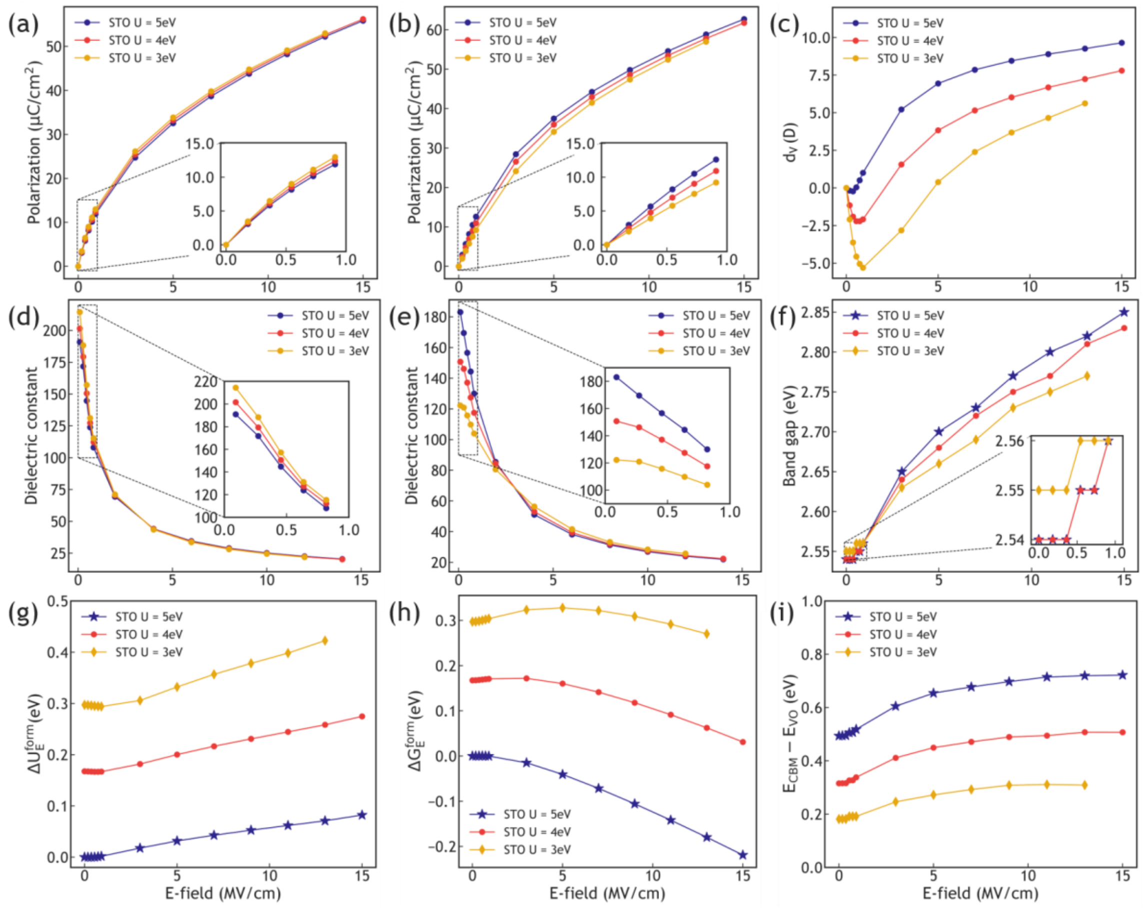

Figure S9. Hubbard U value influence on important physical properties of perfect and defected STO. Polarization of (a) perfect STO, (b) defected STO, (c) d_V, dielectric constants for (d) perfect STO (e) defected STO, (f) band gaps for defected STO, (g) relative formation energies, (h) relative electric Gibbs free energies and (i) $E_{CBM} - E_{VO}$ with different Hubbard U values as functions of electric field. Relative formation energies and electric Gibbs free energies were plotted using the formation energy and electric Gibbs free energy of U = 5eV under zero field strength as reference point (zero). All yellow, red, and blue curves correspond to Hubbard U values equal to 3eV, 4eV and 5eV, respectively.

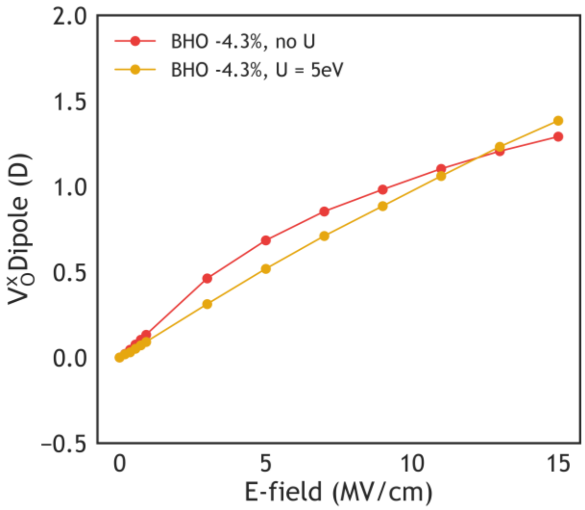

Figure S 10. Oxygen vacancy dipole moment with respect to electric field for $BaHfO_3$ under compression with local Hubbard U correction being 5eV (yellow) and no correction (red).

b) Role of d_V in defect formation energy

With local Hubbard U method, we can reliably obtain the defect formation energy of neutral oxygen vacancy under electric field. Recall Equation (1) from the main manuscript:

$$G_E^{form} = (U^{def} - U^{perf} + \mu_O) - \mathrm{V}\vec{E} \cdot (\vec{P}^{def} - \vec{P}^{perf}) \quad (1)$$

where U and P are the internal energy and polarization of defect-free (perf) or defected (def) SrTiO3 under electric field, respectively, μ_O is the chemical potential of oxygen, V is the volume of the supercell, and $\vec{E}$ is the electric field. The first part of the equation represents the relative internal energy of the system under electric field, and the second part of the equation represents the dot product of oxygen vacancy dipole moment, d_V, and electric field.

$$d_V = \mathrm{V} \cdot \left(\vec{P}^{def} - \vec{P}^{perf}\right) \quad (2)$$

In Figure S11, we have shown the relative Gibbs free energy along with its two components, relative internal energy and the dot product of d_V and electric field. As internal energy increased relatively under electric field, the increase in d_V, which resulting in the decrease in relative Gibbs free energy, dominated over the internal energy change. Therefore, d_V is the main term determining the Gibbs free energy of neutral oxygen vacancy under electric field.

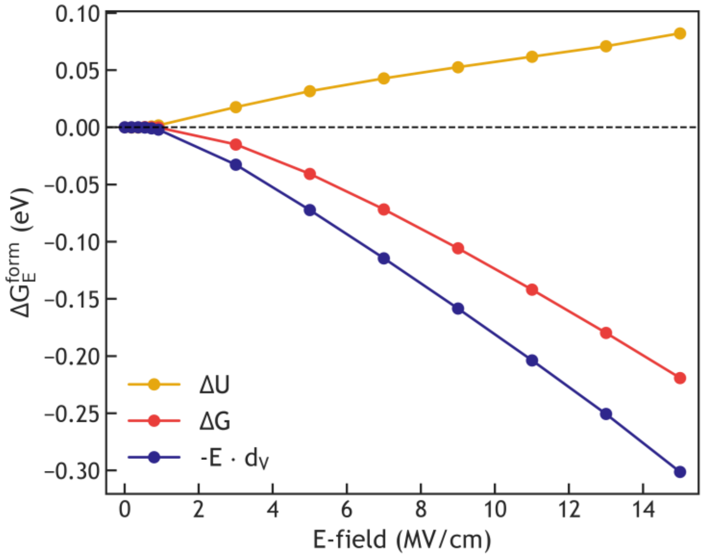

Figure S11 Relative electric Gibbs free energy for neutral oxygen vacancy in STO with field applied parallel to $Ti^{4+} - V_O^x - Ti^{4+}$ chain (red) with two components: relative internal energy (yellow) and $E \cdot d_V$ (blue) which increase and decrease relative electric Gibbs free energy, respectively.

3. Strain effect on dielectric and electronic properties in STO

Strain is known to influence the dielectric constant of materials significantly. In Figure S12, we have shown the strain effect on dielectric constant, polarization, bandgap, and $E_{CBM} - E_{VO}$ with respect to electric field in STO. It is clear that tensile strain could increase the dielectric constant of STO significantly under low field, but the dielectric constant also dropped rapidly with respect to the field strength. Bandgap is known to have an inverse relationship with dielectric constant[38]. In Figure S12(c), we have also demonstrated the strain effect on bandgap, where tensile strain decreased the bandgap of STO while increasing its dielectric constant.

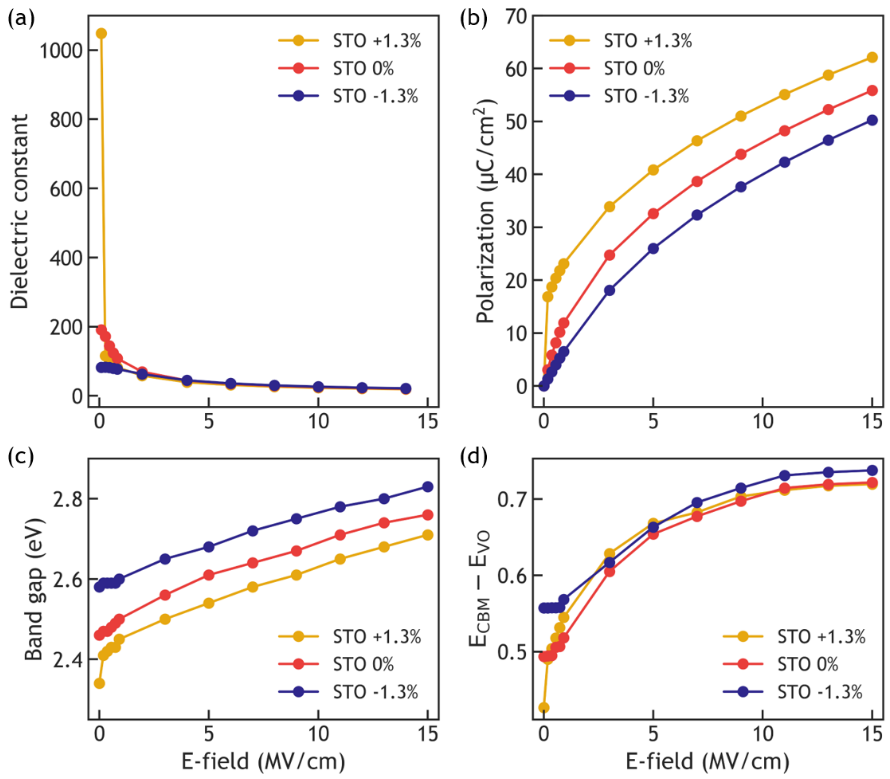

Figure S12. Strain effect on the (a) dielectric constant, (b) polarization, (c) bandgap, and (c) $E_{CBM} - E_{VO}$ with respect to electric field in STO.

4. Electric field direction perpendicular to $Ti^{4+} - V_O^x - Ti^{4+}$ chain – octahedral rotation

When electric field was applied perpendicular to $Ti^{4+} - V_O^x - Ti^{4+}$ chain, defected STO started to show distorted structure at around 0.4MV/cm as shown in Figure S13. In all our calculations, we enforced cubic symmetry on all compounds. However, STO has tetragonal phase as stable structure below 105K[13]. Therefore, we believe that by introducing an oxygen vacancy, and applying the electric field along the undesirable direction (perpendicular to $Ti^{4+} - V_O^x - Ti^{4+}$ chain for STO) on defected STO, we could induce this structure distortion/phase transition. We then used the distorted structure from the 0.4MV/cm calculation, and decreased the field down to zero. We found that the crystal structure remained distorted even when it was fully relaxed under zero field, and assumed a polar phase. Although no such phase change/structure distortion was found in perfect STO under any field strength, we believe it would not be meaningful nor physical to compare the physical properties of the perfect cell with that of the defected cells in different phases. Therefore, we manually constructed a distorted perfect STO structure, by adding one oxygen ion into the distorted defected STO which was fully relaxed under zero field, and allowed the perfect structure to fully relax under zero field again. We then conducted the same process for electric field calculations on this distorted perfect STO. For the data analysis, since defected STO started to show distorted structure when electric field exceeded 0.4MV/cm, we also used the distorted perfect STO to calculate the formation energies and d_V for field strength higher than 0.4MV/cm, and selected the normal perfect STO for field strength smaller than 0.4MV/cm. Therefore, there was a “stitch” at field strength 0.4MV/cm, which indicated the boundary between normal and distorted structures. In Figure S14, we have shown the relative electric Gibbs free energies and d_V curves with stitching, where the distorted perfect structure was used when field exceeded 0.4MV/cm, and without stitching, where the normal perfect structure was used throughout all fields. For the d_V, it is clear that all trends are consistent between two curves, except for the absolute values of the curves, and the kink in the stitched curve is smaller. For the relative electric Gibbs free energies, the trends between two curves are also very close, and the different directions of the kink at 0.4MV/cm originated from the different formation energies (U) for different structures.

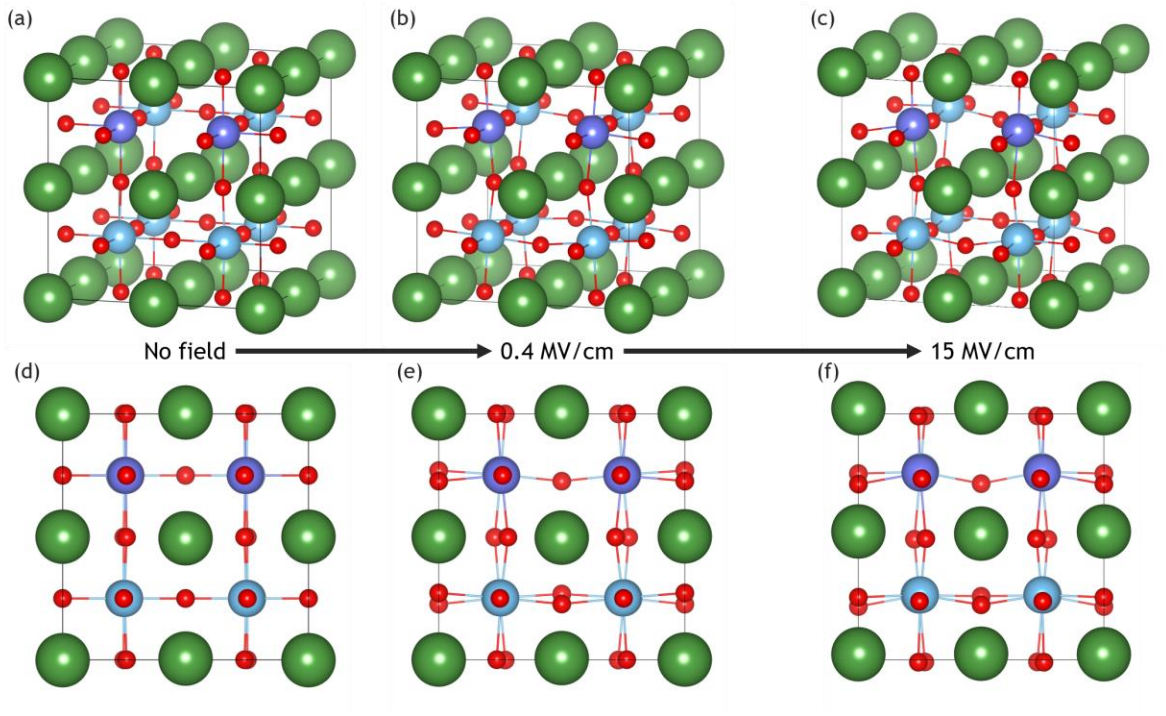

Figure S13. Crystal structures of STO with electric field applied perpendicular to $Ti^{4+} - V_o^x - Ti^{4+}$ chain. STO showed (a) undistorted structure under zero field strength, and transformed into (b) distorted structure starting from 0.4MV/cm, continue to the highest field strength (c) 15MV/cm. (d), (e) and (f) are the side projected views for (a), (b) and (c), respectively.

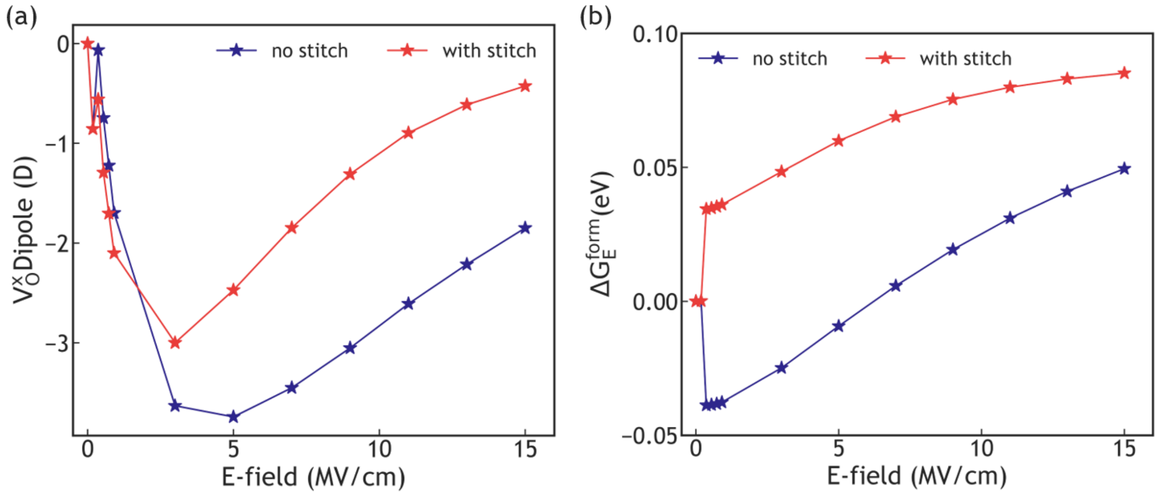

Figure S14. (a) d_V and (b) relative electric Gibbs free energies curves using different perfect STO structures for fields above 0.4MV/cm. For curves with stitching, distorted perfect structure was use when field exceeded 0.4MV/cm, to match the distorted defected structure. For curves with no stitching, normal perfect structure was used throughout all field strengths.

5. Wannier calculation details and site-decomposed polarization

To trace the site polarization difference between a defected and perfect cell, we utilized maximally localized Wannier functions[39,40] with WANNIER90 version 3.1[41] to obtain the site-decomposed polarization from our Berry phase results generated from PWSCF, Quantum Espresso[3,4]. We only conducted Wannier calculations on STO and BHO since we believed these two compounds could already represent the trends for BTO and BZO, respectively. Similar to our previous work[24], we used s-type Gaussians as our initial guess, and allowed projection centers to be guiding centers during the Wannierisation routine (guiding_centres = T). We also found that by increasing the density of the k-point grid, the accuracy for Wannier centers' position could be significantly improved under low fields, while not affecting the Berry phase results, as shown in Figure S6. Therefore, we used denser k-point grids for our Wannier calculations, 2x3x2 k-point grid for field applied parallel to $B^{4+} - V_o^x - B^{4+}$ chain, and 2x2x3 k-point grid for field applied perpendicular to $B^{4+} - V_o^x - B^{4+}$ chain. We selected the MLWFs numbers by targeting the valence bands in perfect structures, and both the valence and defect states in defected structures. We set our convergence threshold on Ω (spread of Wannier functions) to be 5x10^{-8} (conv_tol = 5.0E-8) within two successive iterations (conv_window = 2). We showed the defected STO under zero field strength with Wannier centers in Figure S15.

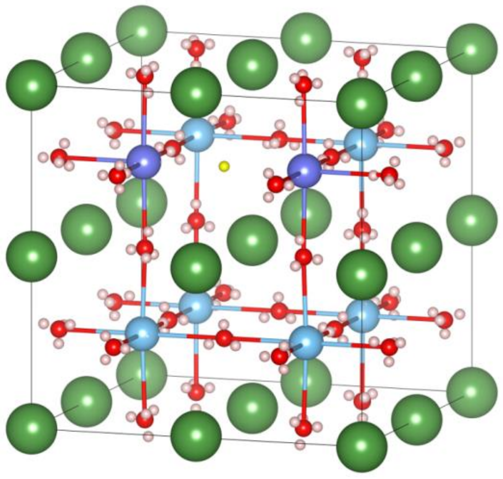

Figure S15. Visualization of Wannier centers in defected STO under zero field strength. Light pink spheres represent the Wannier centers for each electron pair, and the yellow sphere represents the Wannier center of the trapped electrons in the vacant site.

a) Site-decomposed polarization – STO and BHO

In our study, we did not consider electron spin, and the Wannier centers can be viewed as the expectation values of position in real space for each electron pair. As shown in Figure S16, the Wannier centers of the trapped electrons (yellow cross) always had larger displacement than the corresponding oxygen in the perfect cell under all field strengths. The significant displacement difference between trapped electrons and the corresponding oxygen site is the main source promoting d_V when the field is applied parallel to the $Ti^{4+} - V_o^x - Ti^{4+}$ chain. Nevertheless, in contrast to our previous study of alkaline binary oxides, the vacant site was not the only contribution to the d_V in STO. As shown in Figure S16, the approaching Ti ion (left) and the second nearest oxygen ion along the field direction also played an important role in making the defected cell more polarizable. The approaching Ti ion is highly polarizable under the field due to the high reducibility of Ti ions and the attraction from the neighboring trapped electrons, as discussed in the main manuscript. For the oxygen ion, although the displacements of this site are similar in the defected and perfect cells, the circled Wannier center of this oxygen site in the defect cell has relatively large displacement compared to the all other Wannier centers of the corresponding site in the perfect cell, resulting in a larger dipole moment. This may result from the large displacement of the trapped electrons in the vacant site under the field, which repelled the electron cloud of the oxygen. The repulsion would additionally assist the oxygen and its Wannier centers being polarized away from the Ti ion, leading to larger dipole moment. On the other hand, the receding Ti ion (right) in the defected cell showed comparable displacement to the corresponding site in the perfect cell under all field strengths, resulting in much smaller influence on d_V compared to the approaching Ti ion. We believed that as the right Ti ion receded further away from the trapped electrons under electric field, the attraction between electrons and Ti ion impeded the movement of the receding Ti ion, which made it more inert and showed comparable displacement to the corresponding Ti ion in the perfect cell. Therefore, only the approaching (left) Ti ion would form a strong dipole moment along with the trapped electrons in the vacant site.

We have also shown the polarization curves (perfect and defected cell) and Gibbs free energy of formation with two different methods, Berry phase and Wannier functions, for field applied parallel (Figure S17) and perpendicular (Figure S18) to the $Ti^{4+} - V_o^x - Ti^{4+}$ chain in STO. It is

clear that with denser k-point grids, the difference in polarization and Gibbs free energy of formation between the Berry phase approach and Wannier function is negligible even under low field.

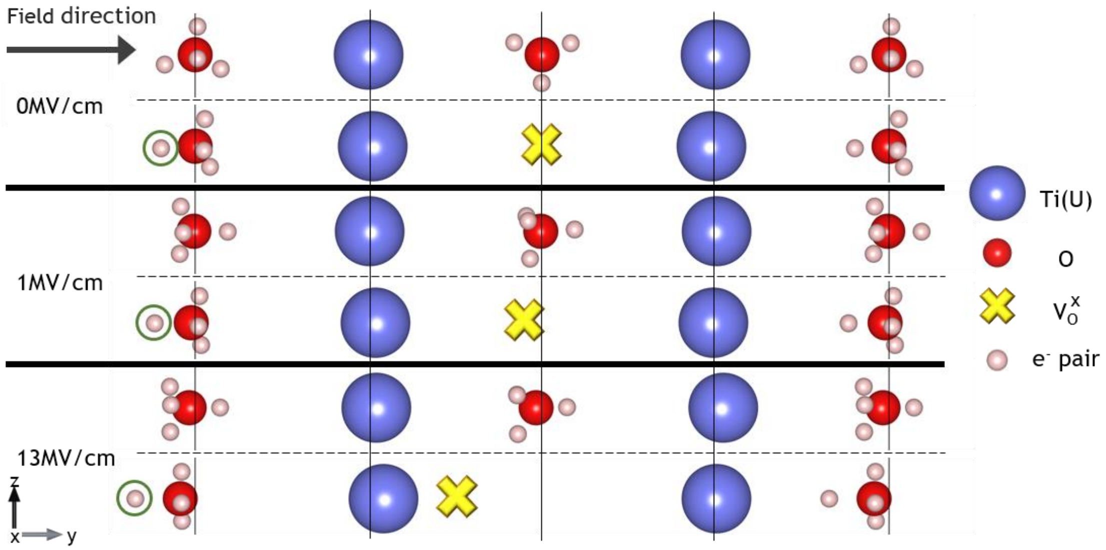

Figure S16. Visualization of the relative displacement between trapped electrons in the vacant site and the corresponding oxygen in the perfect structure from 0MV/cm to 13MV/cm in defected STO. Yellow cross and light pink spheres represent the Wannier centers of the trapped electrons and oxygen electron pairs, respectively. The oxygen ions with circled Wannier centers contributed to the d_V comparably to the trapped electrons in the vacant site. Solid vertical lines are shown to facilitate comparison of displacements, and horizontal dashed lines separate defected and perfect atom chains. Electron pairs (Wannier centers) on Ti ions are not shown.

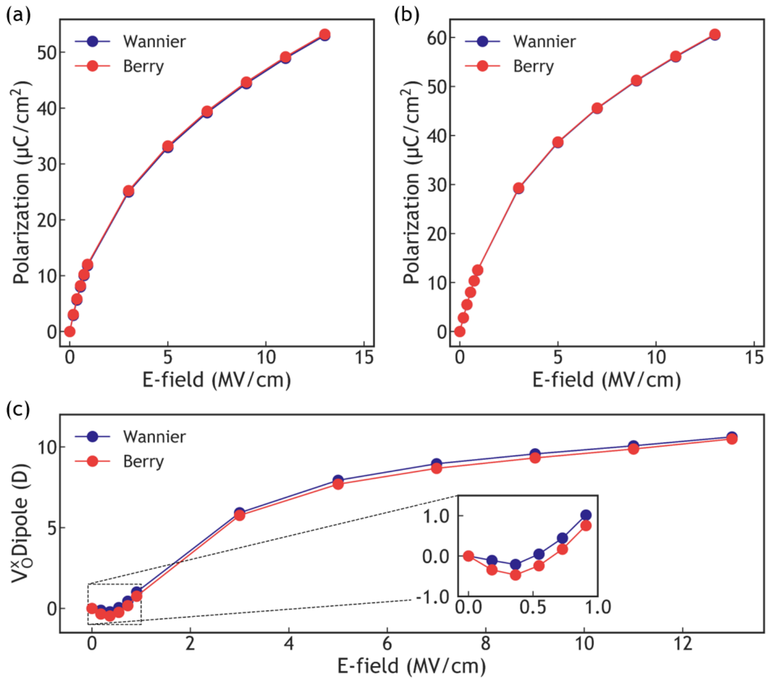

Figure S17. Comparison between Berry phase approach and Wannier function for field applied parallel to $Ti^{4+} - V_o^x - Ti^{4+}$ chain in STO. Polarization curves for (a) perfect and (b) defected STO. (c) d_V curves.

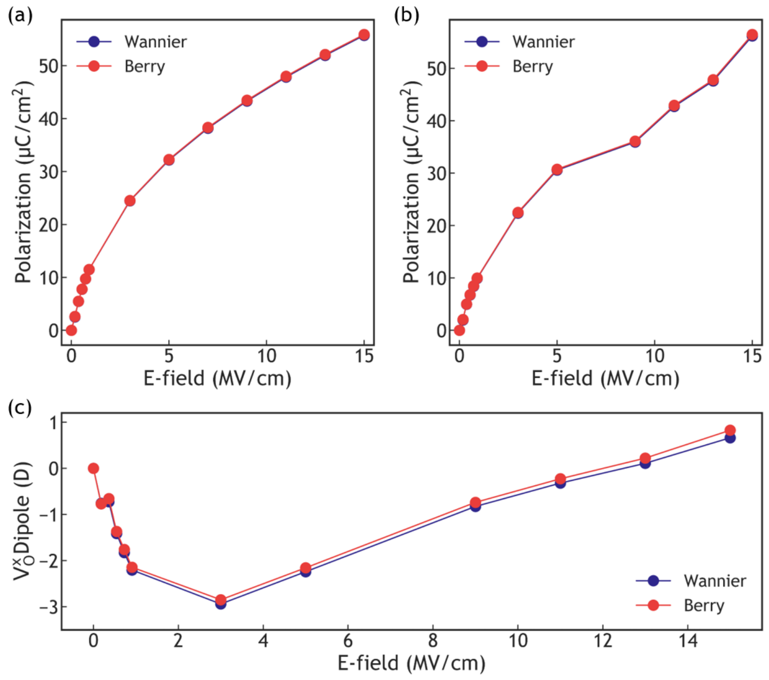

Figure S18. Comparison between Berry phase approach and Wannier function for field applied perpendicular to $Ti^{4+} - V_o^x - Ti^{4+}$ chain in STO. Polarization curves for (a) perfect and (b) defected STO. (c) d_V curves.

To better understand the origin of polarization difference between defected and perfect system, we conducted site decomposed polarization analysis. For instance, in $SrTiO_3$, each ion core was taken as a point charge with charge value based on the pseudopotential, Sr = +10, Ti = +12, O = +6; the Wannier centers were also taken as point charges with charge value = -2 since each Wannier center represented one electron pair. The dipole moment of each point charge under electric field was calculated based on the displacement with respect to their positions under zero field. Following, we assigned Wannier centers (electrons) to their closest ions for clearer definition of "site" dipole, which can be done in this family of oxides because of the relatively clear ionic nature of these oxides. By assigning each Wannier center to the corresponding ion under each field strength, we obtained the field induced polarization/dipole moment for each site in both perfect and defected STO. Subsequently, we can calculate the difference in field induced polarization/dipole moment between the corresponding sites in defected and perfect cell, and constructed the polarization/dipole moment difference curves for each site. d_V can be further expressed as:

$$d_V = d_{def} - d_{perf} = \left[\sum_{i=1}^{N-1} \left(q_{i,def} \Delta\vec{r}_{i,def}\right) + 2e\Delta\vec{r_e}\right] - \left[\sum_{i=1}^{N} \left(q_{i,perf} \Delta\vec{r}_{i,perf}\right)\right] \quad (3)$$

where d_{perf} and d_{def} are the field induced dipole moment of defect-free (*perf*) or defected (*def*) cell, N is the number of ions in the system, q_i and $\Delta\vec{r_i}$ are the charge and the field induced displacement of ion i including its Wannier centers in the direction of the electric field, respectively, e is the electron charge, and $\Delta\vec{r_e}$ is the field induced displacement of the Wannier center of the trapped electrons in the direction of the electric field. Since the dipole moment value for any charge species (e.g. individual charged ion) depends on the observation point, we set the origin points for each site to be their positions under zero field, and calculate the displacement accordingly ($\Delta\vec{r}_i$). Note that the core charges for Ti, Zr and Hf are all +12, where their valence electrons are in orbitals $3s^2 3p^6 4s^2 3d^2$, $4s^2 4p^6 5s^2 4d^2$ and $5s^2 5p^6 6s^2 5d^2$, respectively.

In the following paragraphs, we will use *site dipole* to represent the dipole moment difference of one site in the defected cell with its corresponding site in the perfect cell under finite electric field ($d_{site} = q_{site,def}\Delta\vec{r}_{site,def} - q_{site,perf}\Delta\vec{r}_{site,perf}$). From the site dipole plots, we can directly identify which sites promoted the d_V the most (positive sites), and which sites showed

negative influence (negative sites) on the d_V. As shown in Figure S19, for electric field applied parallel to the $Ti^{4+} - V_O^x - Ti^{4+}$ chain in STO, ions Ti7, Ti8, O17, and the vacant site showed the most positive contribution (positive sites) to the d_V under low field, while only Ti7, O17, and the vacant site remained positive sites under high field. We found that around 75% of d_V is composed of Ti7, O17 and the vacant site, where the contribution percentage for each site was calculated based on the absolute value of d_{site} divided by the sum of the absolute value of d_{site}. Absolute value of d_{site} was used to address the ambiguity of positive and negative contribution. Most other sites showed either negative or insignificant influence on the d_V, and it was clear that Ti7, O17, and the vacant site formed a strong defect-cluster dipole under high field, which dominated the d_V in STO. For electric field applied perpendicular to the $Ti^{4+} - V_O^x - Ti^{4+}$ chain in STO, the definition of positive and negative sites became more complicated. Since there was an octahedral rotation happening around 0.6MV/cm, there might be a sign change of the site dipole at that field strength. Therefore, we defined the negative and positive sites not as the absolute value of the site dipole, but according to whether the sites increase (positive sites) or decrease (negative sites) the d_V. If the site dipole increases with respect to the field, then we define them to be positive sites, and vice versa. As shown in Figure S20, actually all of the ions showed either negative or insignificant influence on the d_V, and only the vacant site acted as the positive site. Nevertheless, the positive effect from the vacant site was still extremely weak under low field (+0.07 D from 0.4MV/cm to 1MV/cm) compared to the vacant site in the parallel case (+0.6 D from 0.4MV/cm to 1MV/cm). We have also highlighted the important positive and negative sites in Figure S 21. These site dipole comparisons in STO clearly explained the origin of different d_V response with respect to electric field with different electric field direction.

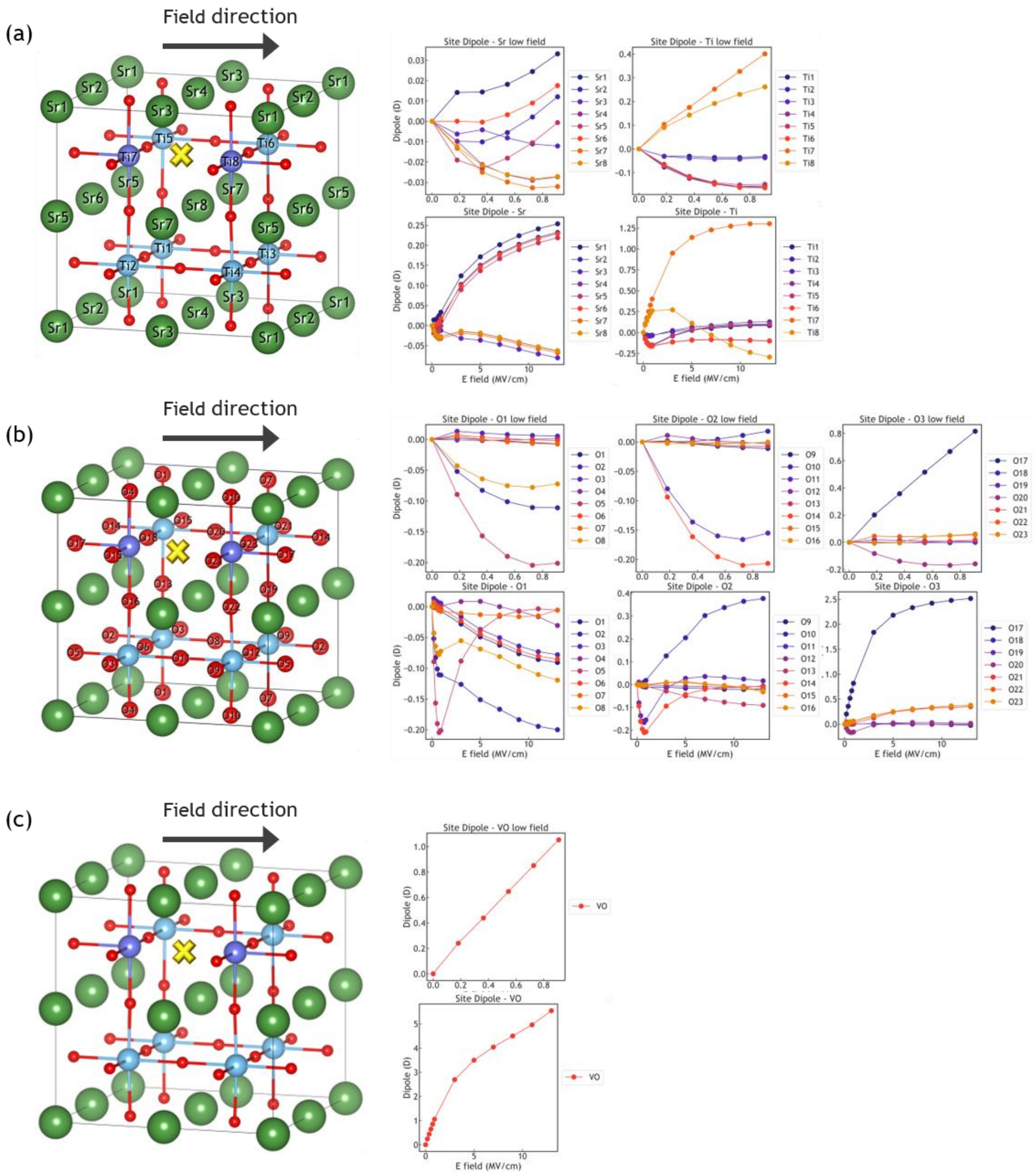

Figure S19. Site dipole moment difference between defected and perfect STO with electric field applied parallel to $Ti^{4+} - V_o^x - Ti^{4+}$ chain for (a) Sr and Ti ions, (b) O ions, and (c) the vacant site.

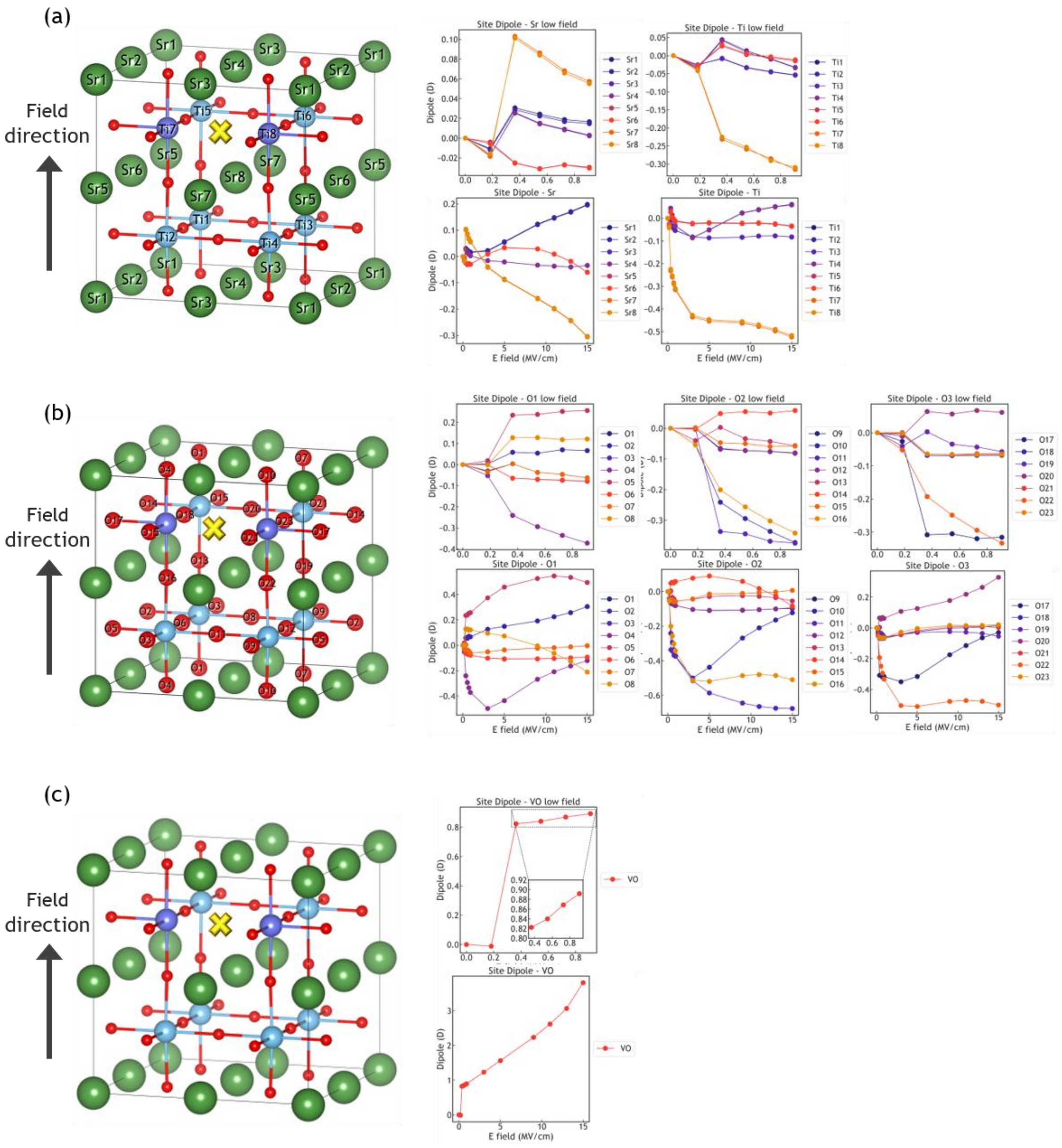

Figure S20. Site dipole moment difference between defected and perfect STO with electric field applied perpendicular to $Ti^{4+} - V_O^x - Ti^{4+}$ chain for (a) Sr and Ti ions, (b) O ions, and (c) the vacant site. The distorted structures were used for both perfect and defected STO after field strength 0.6MV/cm.

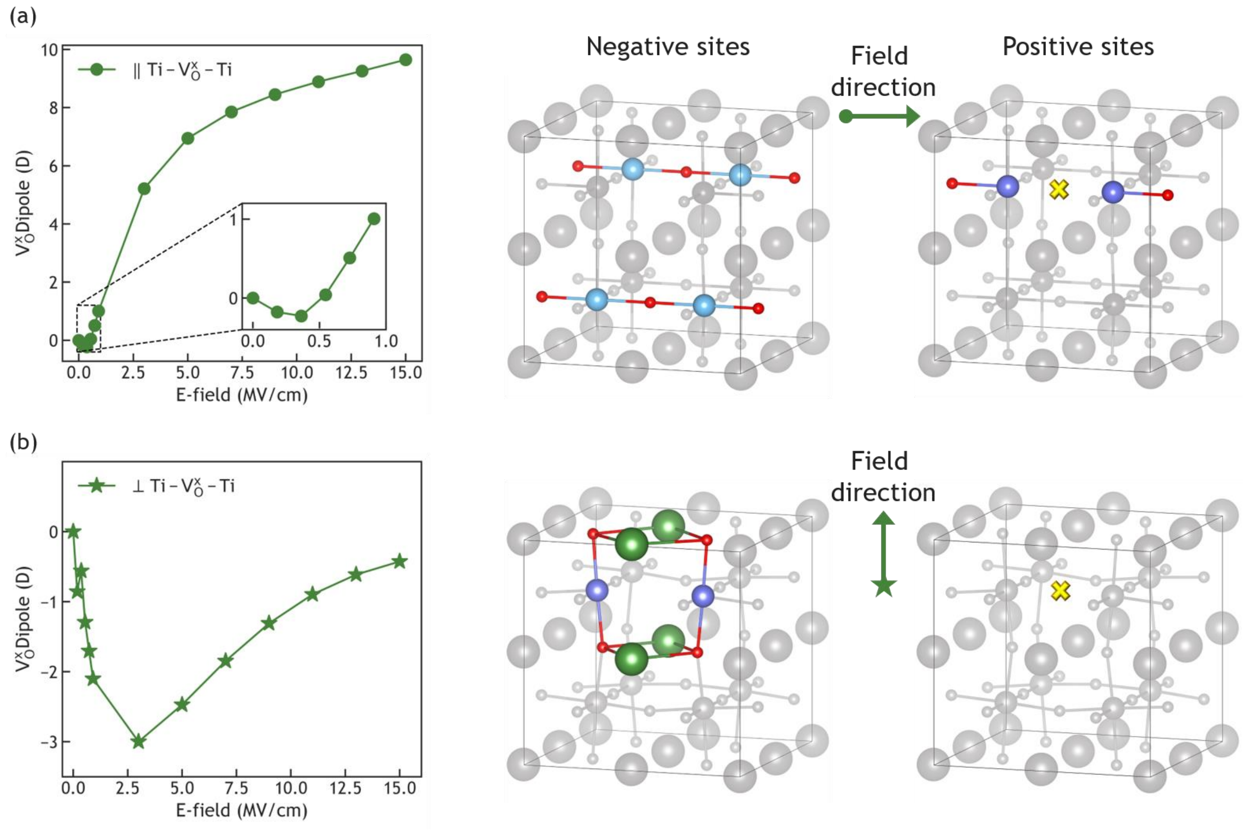

Figure S 21. Oxygen vacancy dipole moment (d_V) in units of Debye (D) and site-decomposed contribution with field applied (a) parallel to the $Ti^{4+} - V_o^x - Ti^{4+}$ chain and (b) perpendicular to the $Ti^{4+} - V_o^x - Ti^{4+}$chain, with the sites which decreased (negative sites) or increased (positive sites) the d_V highlighted. Only the ions with significant contribution are highlighted here.

We also showed the comparison between the Berry phase approach and Wannier function for BHO below with electric field applied parallel and perpendicular to $Hf^{4+} - V_o^x - Hf^{4+}$ chain in Figure S22 and Figure S23, respectively. It is clear that the differences between the two methods are still very small under all fields, and the deviation in the d_V plots seemed more obvious only because of the small d_V in BHO. In Figure S24 and Figure S25, we also showed the site dipole between perfect and defected BHO. In contrast to STO, the site dipole in BHO is very small for each site, and the d_V curves basically followed the trend of vacant site dipole curves. Furthermore, in BHO, the vacant site dipole response to electric field was also opposite to that in STO. For field applied parallel to the $Hf^{4+} - V_o^x - Hf^{4+}$ chain, the vacant site had lower dipole moment than the corresponding oxygen under all electric field strength, resulting in a negative value in the site dipole as shown in Figure S24(c). When field was applied perpendicular to the $Hf^{4+} - V_o^x - Hf^{4+}$ chain, the vacant site became more polarizable, showing a positive value in the site dipole moment difference. We believe the vacant site dipole difference between STO and BHO mainly originated from the different redox ability/electron affinity/electron negativity between Ti and Hf ions, which affected the tendency of electrons to polarize toward or away from the neighboring cations.

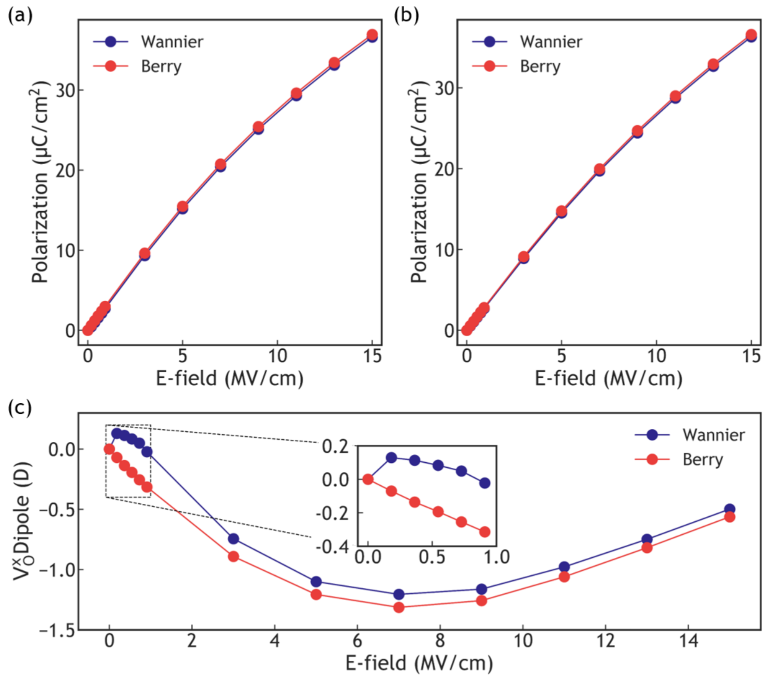

Figure S22. Comparison between Berry phase approach and Wannier function for field applied parallel to $Hf^{4+} - V_o^x - Hf^{4+}$ chain in BHO. Polarization curves for (a) perfect and (b) defected BHO. (c) d_V curves.

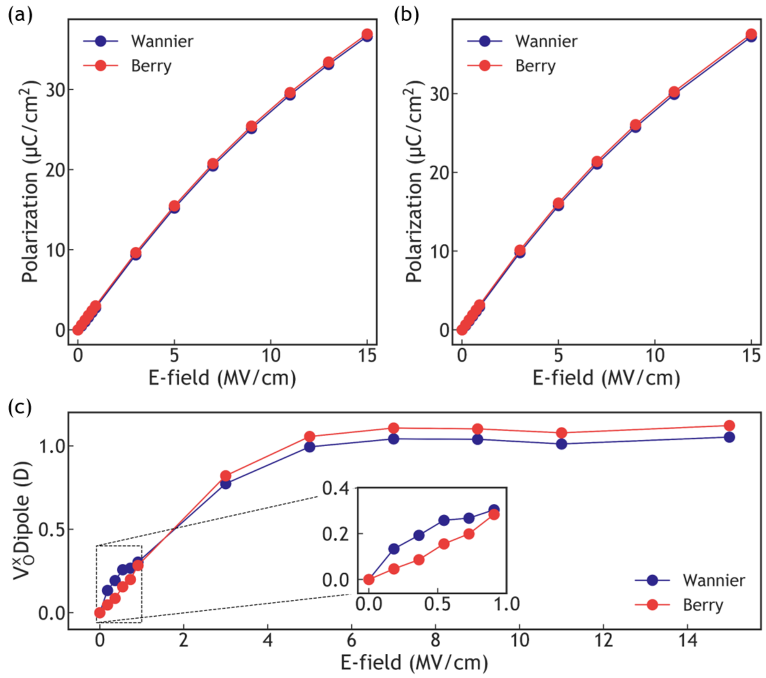

Figure S23. Comparison between Berry phase approach and Wannier function for field applied perpendicular to $Hf^{4+} - V_o^x - Hf^{4+}$ chain in BHO. Polarization curves for (a) perfect and (b) defected BHO. (c) d_V curves.

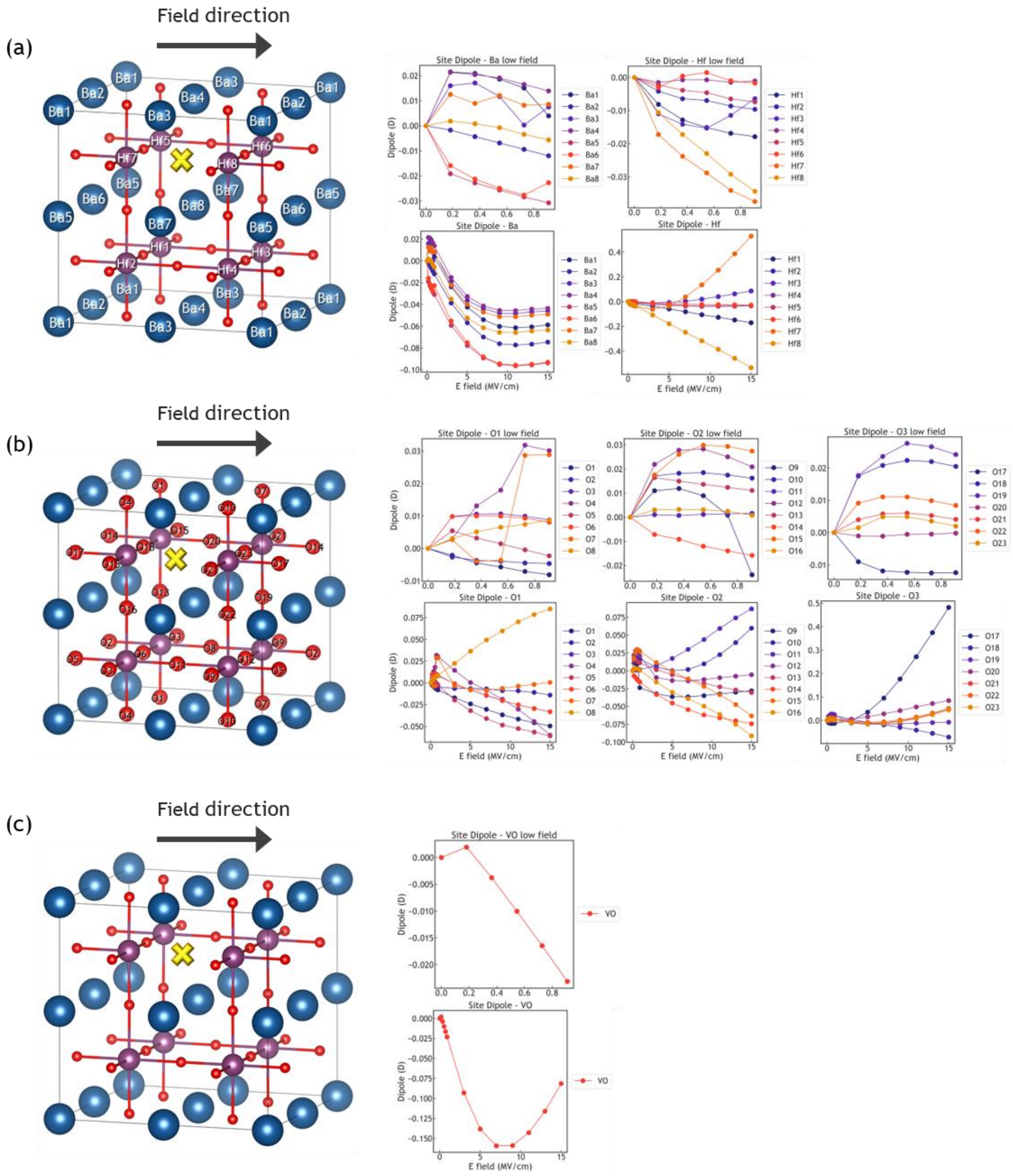

Figure S24. Site dipole moment difference between defected and perfect BHO with electric field applied parallel to $Hf^{4+} - V_o^x - Hf^{4+}$ chain for (a) Ba and Hf ions, (b) O ions, and (c) the vacant site.

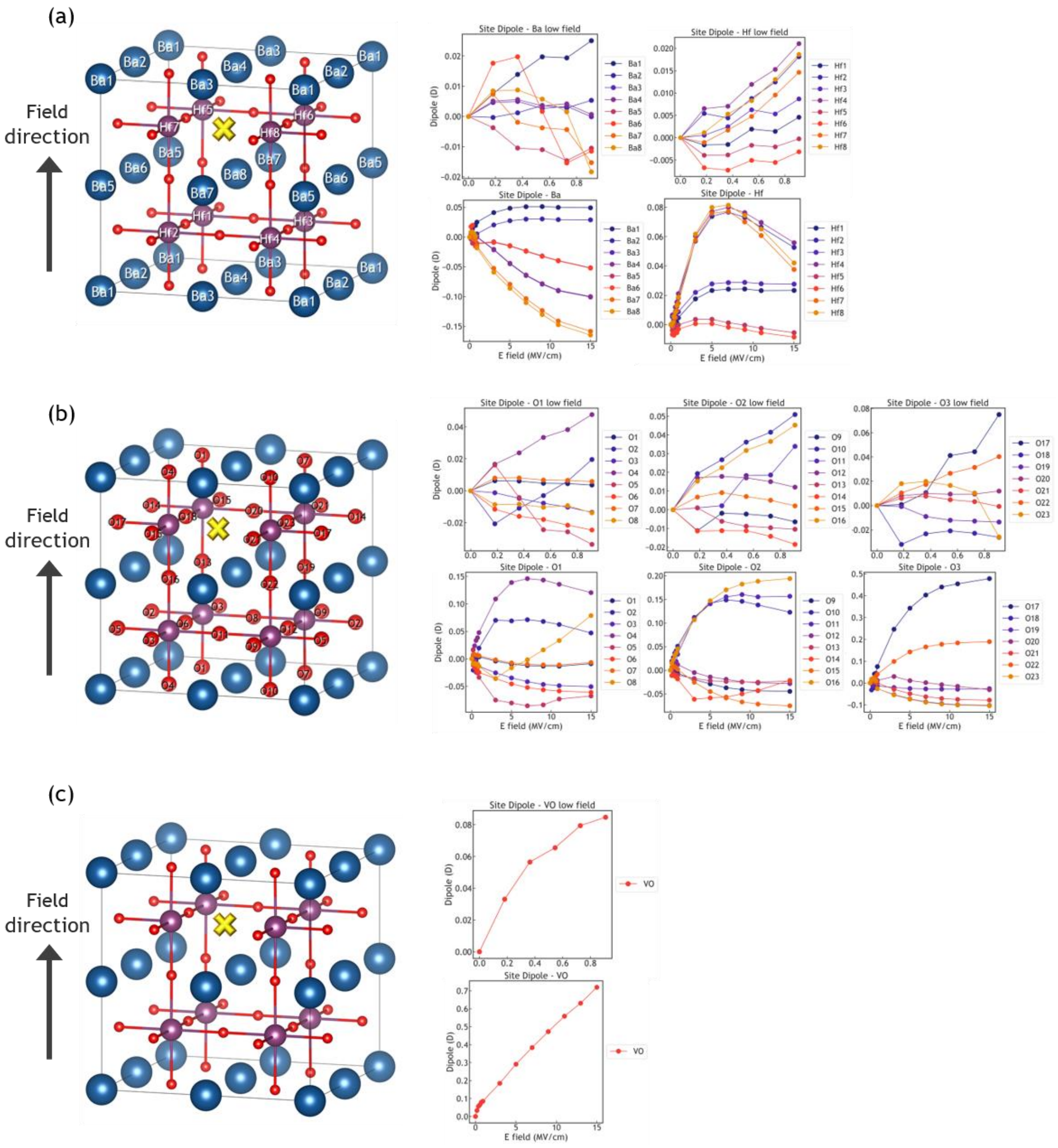

Figure S25. Site dipole moment difference between defected and perfect BHO with electric field applied perpendicular to $Hf^{4+} - V_o^x - Hf^{4+}$ chain for (a) Ba and Hf ions, (b) O ions, and (c) the vacant site.

b) Compressive strain effect on negative d_V under low field

As discussed in section (a), several sites in the defected cell became less polarizable than the corresponding sites in the perfect cell, which contributed negatively to d_V (negative sites). This phenomenon is more prominent under low field strength, and compressive strain played an important role in affecting it, which caused the slight difference in d_V between compressed 2.1% BTO (always positive) and unstrained STO (small negative value). Compressive strain is known to decrease the polarization of ions[42]. Therefore, under compressive strain, the ions in both defected and perfect cell would all became less polarizable, and eventually become almost immobile under extreme compressive strain, where we can see them being inert to external electric field. In Figure S26 (a) and (b), we showed some negative oxygen sites of BTO under -0.8% and -2.1%, respectively. It is clear that the negative sites became less negative under higher compressive strain in Figure S26 (b). We believe the decrease in polarization of ions in both defected and perfect cell muted the negative d_V under low electric field for compressed 2.1% BTO, while for unstrained STO, shown in Figure S26 (c), still had several prominent negative oxygen sites under low electric field. Therefore, even though BTO under 2.1% compression has identical lattice constant to unstrained STO, the large compressive strain on BTO resulted in the slight difference in d_V between these two systems under low field.

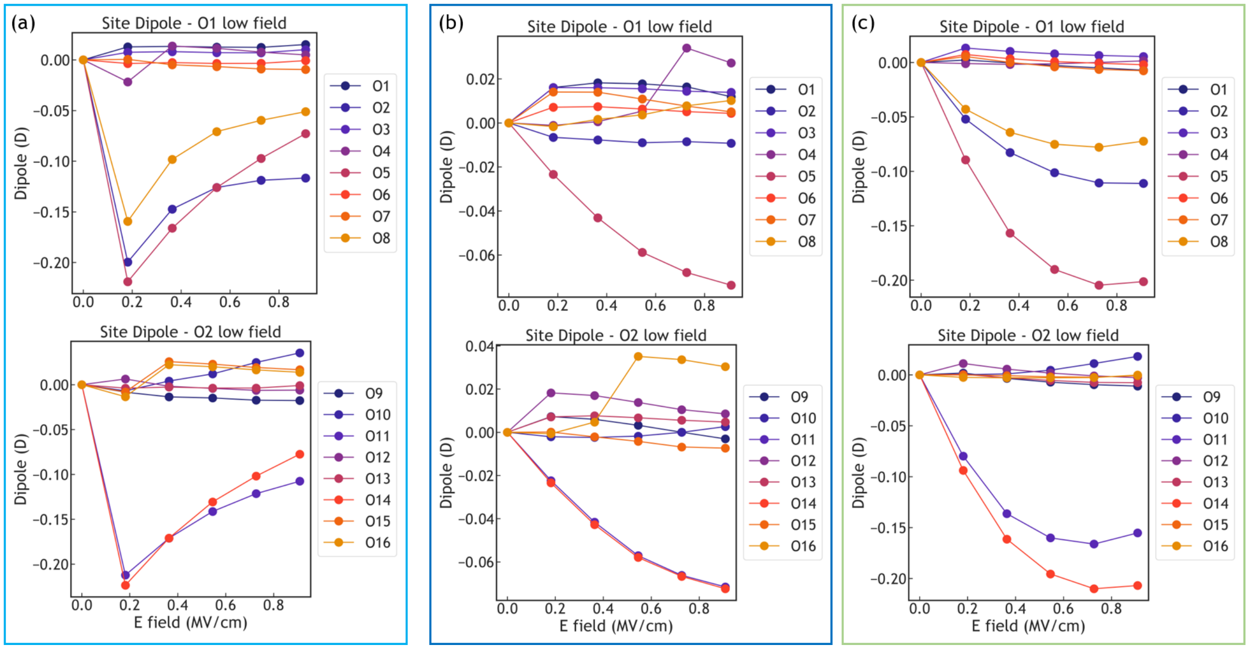

Figure S26. Selected oxygen site dipole moment difference between defected and perfect BTO under (a) 0.8% compression, (b) 2.1% compression. The lattice constant of BTO under 1.3% compression matches the lattice constant of (c) unstrained STO. Electric field was applied parallel to $Ti^{4+} - V_o^x - Ti^{4+}$ chain.

b) Favored polarization direction and strain effect on d_V

As shown in Fig. 3 in the main manuscript, with increasing volume, d_V would become more positive with field applied parallel to the favored polarization direction (parallel to $B^{4+} - V_o^x - B^{4+}$ for STO and BTO, perpendicular to $B^{4+} - V_o^x - B^{4+}$ for BZO and BHO), while become more negative when field was applied perpendicular to the favored polarization direction. In Figure S27, we further showed the strain effect on d_V within the same compound, BZO and BHO. Consistent with our results in Fig. 3, with increasing volume (tensile strain, lighter color lines), d_V became more positive when field was applied parallel to the favored polarization direction, and became more negative when field was applied perpendicular to the favored polarization direction. With decreasing volume (compressive strain, darker color lines), the effect is reversed.

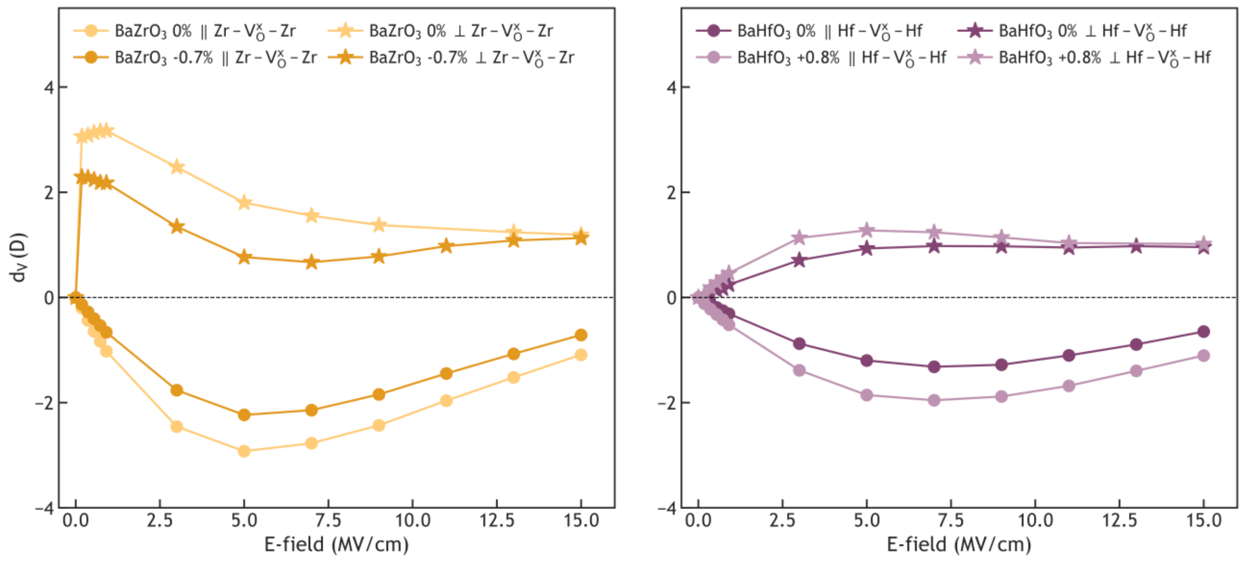

Figure S27 Oxygen vacancy dipole moment for (a) BZO and (b) BHO under different strain states and electric field direction, parallel and perpendicular to $B^{4+} - V_o^x - B^{4+}$ (B = Zr, Hf).

c) Compressive strain promotes d_V in BHO and BZO

As shown in Fig. 3 in the main manuscript, the d_V of BHO and BZO under extreme larger compressive strain went through a sign change, from negative (no strain) to positive (4~5% compressive strain). We believe the origin of the increase of d_V in BHO and BZO under compressive strain, which is opposite to the case in STO and BTO, is owing to such large (and unphysical) compression overcoming the low reducibility of Hf and Zr ions. In Figure S28, we have shown the relative displacement of the $O - Hf^{4+} - V_o^x - Hf^{4+}$ chains in unstrained and compressed BHO under 0MV/cm and 3MV/cm, where the length of the ion chains under different strain states are normalized for better visualization of the fractional displacement of each site. Comparing the ion chains under 0MV/cm with different strain conditions, we can clearly see that the Hf ions under -4.3% strain showed higher tendency relaxing toward the trapped electrons than the ones in unstrained case. Furthermore, under 3MV/cm, the left Hf ions (Hf1) under compression also had larger displacement than the one in unstrained cell. The consequence of having larger displacement under electric field is displaying higher field induced dipole moment.

In Figure S29, we have shown the site dipole and pure dipole – the field induced dipole moment for one site in the defected cell ($d_{site,pure} = q_{site,def}\Delta\vec{r}_{site,def}$) for oxygen vacant site and Hf1 in BHO. Compression suppressed the polarization for both trapped electrons and the corresponding oxygen site in the perfect cell; nevertheless, the suppression on oxygen ion is stronger, making the site dipole for oxygen vacant site less negative, as shown in Figure S29 (a). However, the main source promoting d_V is the Hf1 ion instead of the trapped electrons. As shown in Figure S29 (b), both the site dipole and pure dipole for Hf1 increased under larger compressive strain, where the magnitude of the changes are also much larger than that in oxygen vacant site. Noted that since the reducibility of Hf and Zr ions are much lower than that of Ti ion, the direct contribution of trapped electrons to d_V is less significant than that in STO or BTO, while the influence of B site cation on d_V increased. Nevertheless, the existence of the vacant site as well as the trapped electrons still played an important role in affecting the polarization of the B site cation under electric field. In Figure S30, we also showed the site and pure dipole for Hf2 and O17 under different strain states and electric field strength. The origin of the larger site and pure dipole for Hf2 is also owing to the higher tendency of Hf ions relaxing toward the

trapped electrons under zero field. Under zero field, the Hf2 ion in compressed cell had a more negative (left) starting point to the field direction. Therefore, it can relax more than the corresponding site in the unstrained and perfect cell under electric field, resulting in larger site and pure dipole. We believe the larger site and pure dipole for O17 also resulted from Hf ions leaning toward the oxygen vacant site, which subsequently provided more space for O17 to be polarized under electric field. This observation can also be carried to BZO, where the large compressive strain made the whole $O - B^{4+} - V_o^x - B^{4+}$ chain (B = Hf or Zr) more similar to $O - Ti^{4+} - V_o^x - Ti^{4+}$. In summary, the extremely large compressive strain made the B site cation (B = Hf or Zr) relax more toward the trapped electrons, and subsequently affected the whole $O - B^{4+} - V_o^x - B^{4+}$ chain in BZO and BHO, which promoted d_V and exhibited the sign change.

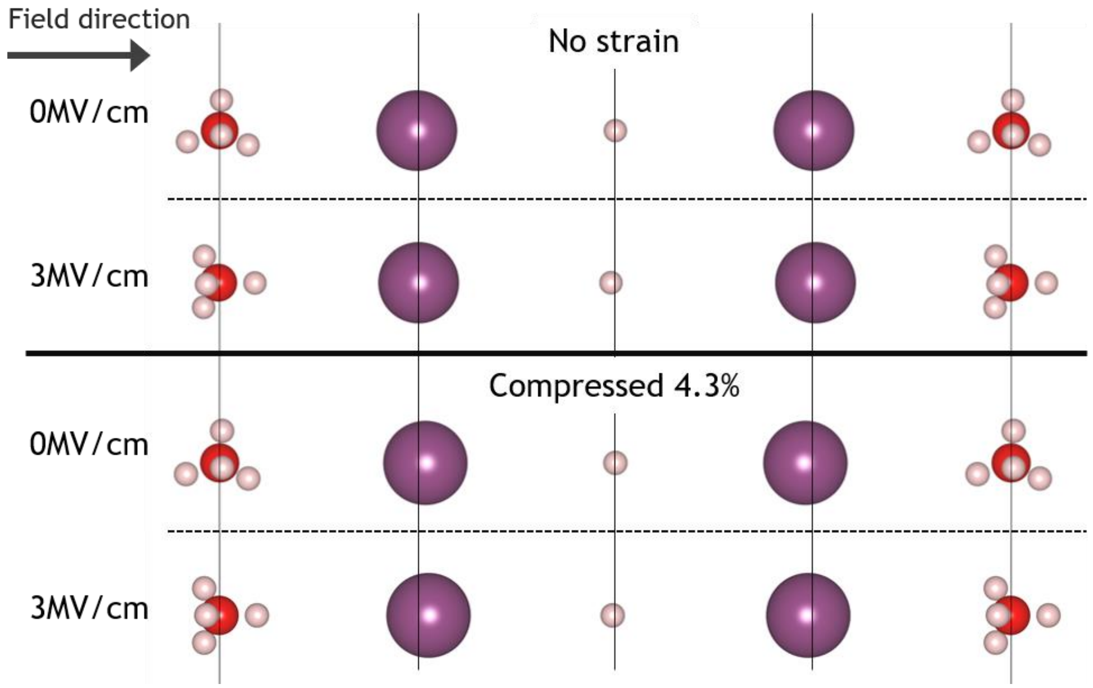

Figure S28 Visualization of the relative displacement of Hf ions, Wannier center of the trapped electrons and the oxygen ion next to the Hf ions (O17) in BHO under no strain (top) and -4.3% strain (bottom). Purple, red and light pink spheres represent Hf, O ions and Wannier centers, respectively. The field strength is shown on the left side of each ion chain, and the black vertical line represent the initial position of each ion under zero field and unstrained condition (position of each ion in the very top ion chain). Length of the ion chains under different strain states are normalized.

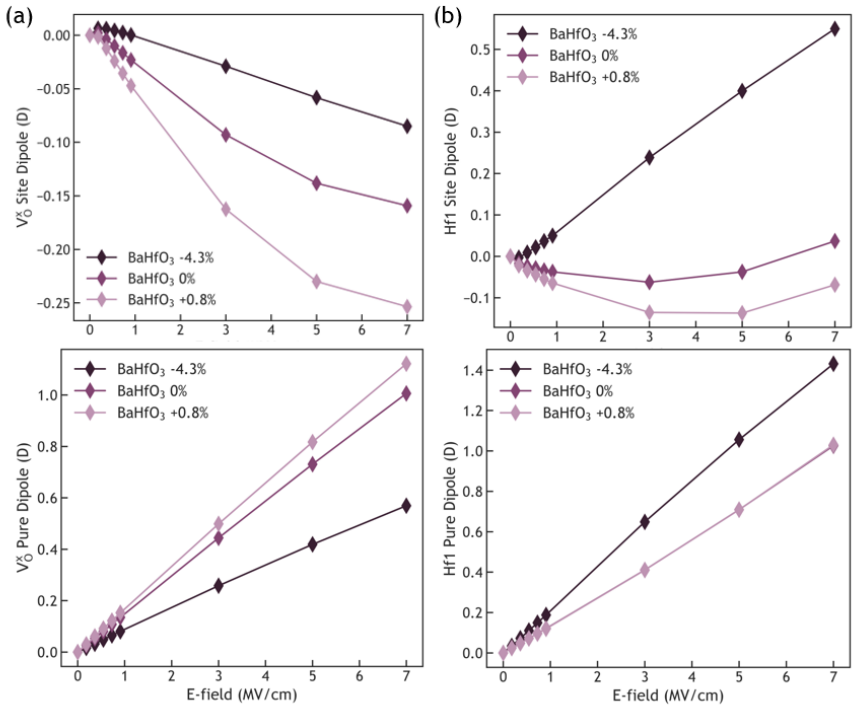

Figure S29 Site dipole (top) and pure dipole (bottom) of (a) oxygen vacant site and (b) Hf1. Electric field was applied parallel to $Hf^{4+} - V_o^x - Hf^{4+}$ chain.

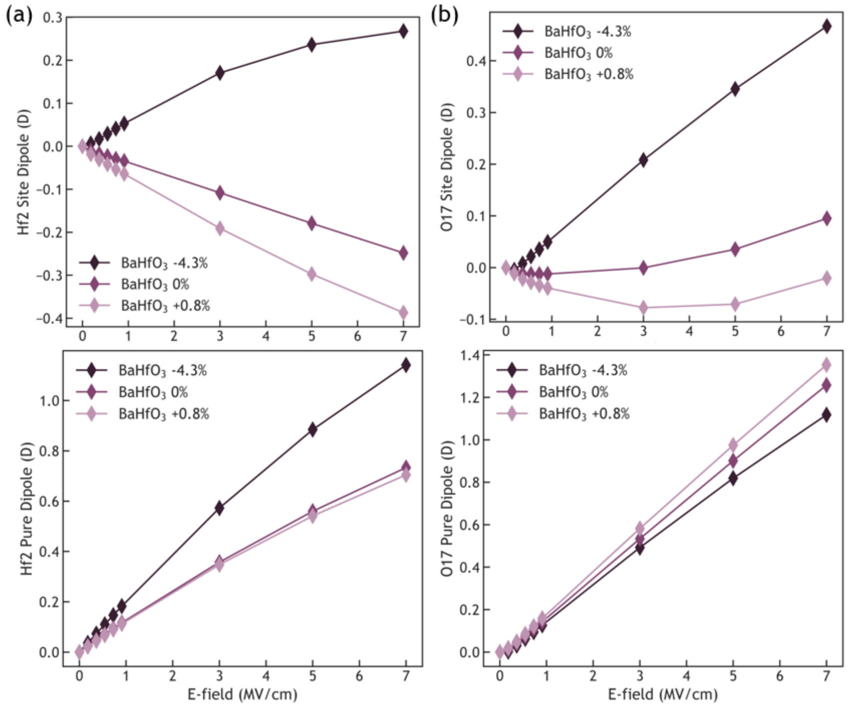

Figure S30 Site dipole (top) and pure dipole (bottom) of (a) Hf2 and (b) O17. Electric field was applied parallel to $Hf^{4+} - V_o^x - Hf^{4+}$ chain.

6. Electronegativity, d_V and d_{B-VO-B},

Although d_V shows a strong positive correlation with the electronegativity of the B site cation (Fig. 4 in the main manuscript), BZO and BHO under small or zero strain do not follow the trend perfectly. This is owing to the fact that d_V represents the net dipole moment difference between the whole defected and perfect cell under electric field, while the B site cation electronegativity can only reflect the extent of electron localization in the defected system. Therefore, the electronegativity of B site cannot fully capture every component in d_V. In this case, why do we still see a strong correlation between B site cation electronegativity to d_V?

Recall the Wannier analyses above, the main sites contributed to d_V are the oxygen vacant site and its neighboring ions. The field induced dipole moment of the main segment ($B^{4+} - V_o^x - B^{4+}$) of this defect cluster, d_{B-VO-B}, can be expressed with:

$$d_{B-VO-B} = q_{B1,def}\Delta\vec{r}_{B1,def} + 2e\Delta\vec{r_e} + q_{B2,def}\Delta\vec{r}_{B2,def} \quad (4)$$

where B1 and B2 represent the B site cation (B = Ti, Zr, Hf) being polarized toward and away from the trapped electrons, respectively. The electronegativity of B site cation can perfectly capture the magnitude of this quantity under electric field, since it reflects the interaction between the trapped electrons and the neighboring B site cation, which determines the response of this defect cluster under electric field. Therefore, with d_{B-VO-B} being the main component, d_V would also show a strong correlation with B site cation electronegativity.

In Figure S31, we have shown the field induced dipole moments for different defect clusters with respect to the B site cation electronegativity. We believe the most adequate defect cluster to directly connect with B site electronegativity is the $B^{4+} - V_o^x - B^{4+}$ chain, as shown in Figure S31 (a). In Figure S31 (b), we can see the field induced dipole moment for single oxygen vacant site does not perfectly correlate with B site cation electronegativity. This discrepancy mainly resulted from that we should not correlate electronegativity with a “single site”, but to connect it to a “bonding state”. The difference in electronegativity between two elements have been widely used to described the covalency of the bond between two different elements. Although there was no bonding between the trapped electrons and the neighboring B site cation, they still affect each other’s polarization simultaneously – a two-way influence. Therefore, it would be unphysical to isolate the oxygen vacant site alone when correlating to B site cation electronegativity; instead,

we should include the whole defect cluster to capture the whole picture. In Figure S31 (c), we have also shown the $O - B^{4+} - V_o^x - B^{4+}$ chain with respect to the B site cation electronegativity. The trends in Figure S31 (a) and (c) are almost identical, and $O - B^{4+} - V_o^x - B^{4+}$ actually contributed to d_V slightly more than $B^{4+} - V_o^x - B^{4+}$ alone. Nevertheless, the oxygen in between the B site cations does not directly interact with the trapped electrons in the vacant site, where it was indirectly affected by them under electric field. Furthermore, the contribution difference between two different defect clusters was also very small. Therefore, in order to have a simpler and more direct connection between the defect cluster dipole moment and B site cation electronegativity, we believe $B^{4+} - V_o^x - B^{4+}$ chain should be the best defect cluster we should select.

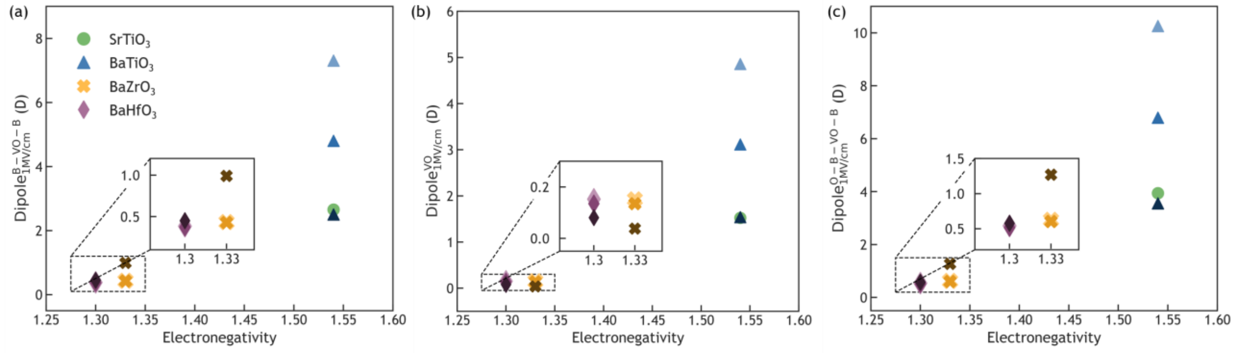

Figure S31. Field induced dipole moment (pure dipole) for (a) $B^{4+} - V_o^x - B^{4+}$ chain, (b) V_o^x site, and (c) $O - B^{4+} - V_o^x - B^{4+}$ chain under 1MV/cm for STO, BTO, BZO and BHO under different strain states. Field was applied parallel to $B^{4+} - V_o^x - B^{4+}$ chain (B = Ti, Zr, Hf).

Other than the electronegativity of the B site cation, we also tried to correlate the defect polarization to a more material-specific quantity - the effective mass of electron at conduction band minimum (CBM). We believe that the more reducible the B site cation (equivalent to higher electronegativity), the more easily it can "trap" the electron, which will lead to larger electron effective mass. Therefore, defect cluster dipole and oxygen vacancy dipole moment should also correlate positively to electron effective mass.

To validate the hypothesis, we calculated the band structures and the corresponding electron effective mass at CBM along two high symmetry reciprocal space directions (Γ to X and Γ to M)[43] for $BaTiO_3$, $BaZrO_3$ and $BaHfO_3$ with all materials having the same lattice constant (lattice constant of unstrained $BaTiO_3$) to exclude the effect of lattice volume. As shown in Figure S32,

the trend of defect cluster dipole and oxygen vacancy dipole moment both correlate positively to electron effective mass regardless of band and k-space direction (Γ to X or Γ to M). Such trend is identical to that being plotted against B site cation electronegativity (Figure 4 in the main manuscript).

Finally, since the d_{B-VO-B} of $BaTiO_3$ and Ba(Zr, Hf)O_3 responded differently to lattice strain, where the former increase with tensile strain, and the latter increase with compressive strain, we did a rough interpolation and found a possible transition region where the strain should have no effect on d_{B-VO-B} around B site cation electronegativity being around 1.4. Interestingly, there is no element having electronegativity being exactly 1.4, where only uranium (U) with electronegativity being 1.38[44] is close enough to fall into this transition region.

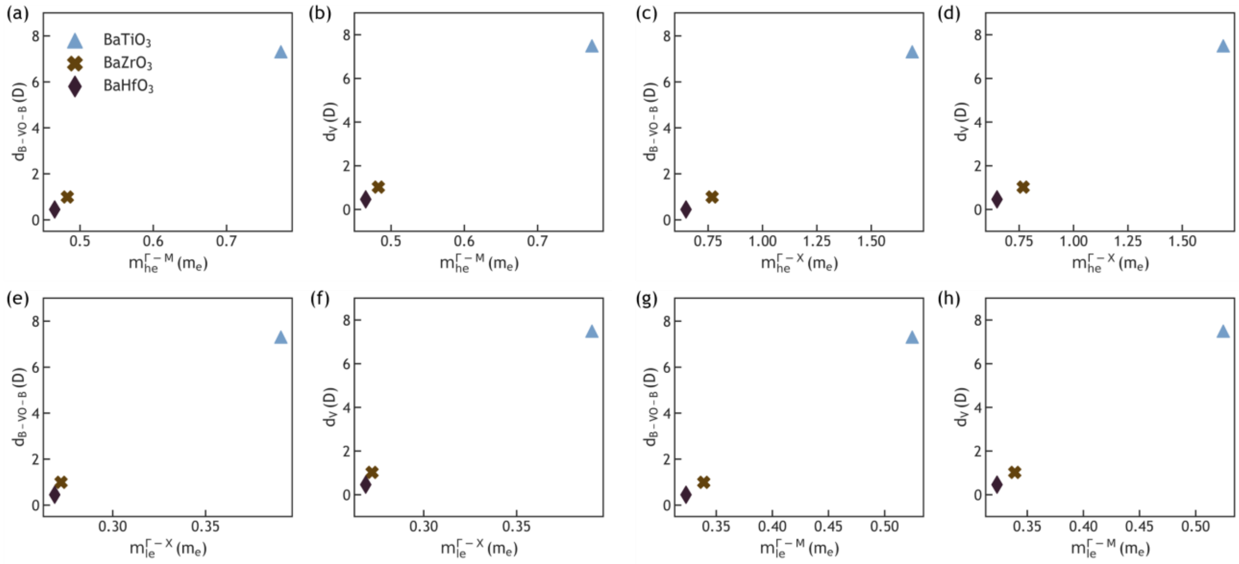

Figure S32. Defect cluster dipole and oxygen vacancy dipole moment with electric field applied parallel to $B^{4+} - V_O^x - B^{4+}$ plotted against electron effective mass at CBM. Electron effective masses were obtained from heavy electron (he) band and light electron (le) band, with two high symmetry directions in reciprocal space, Γ to X and Γ to M[43].

Reference

[1] I. Souza, J. Íñiguez, D. Vanderbilt, *Physical Review Letters* **2002**, *89*, DOI 10.1103/PhysRevLett.89.117602.

[2] N. A. Spaldin, *Journal of Solid State Chemistry* **2012**, *195*, 2.

[3] P. Giannozzi, O. Andreussi, T. Brumme, O. Bunau, M. B. Nardelli, M. Calandra, R. Car, C. Cavazzoni, D. Ceresoli, M. Cococcioni, N. Colonna, I. Carnimeo, A. D. Corso, S. de Gironcoli, P. Delugas, R. A. DiStasio, A. Ferretti, A. Floris, G. Fratesi, G. Fugallo, R. Gebauer, U. Gerstmann, F. Giustino, T. Gorni, J. Jia, M. Kawamura, H.-Y. Ko, A. Kokalj, E. Küçükbenli, M. Lazzeri, M. Marsili, N. Marzari, F. Mauri, N. L. Nguyen, H.-V. Nguyen, A. Otero-de-la-Roza, L. Paulatto, S. Poncé, D. Rocca, R. Sabatini, B. Santra, M. Schlipf, A. P. Seitsonen, A. Smogunov, I. Timrov, T. Thonhauser, P. Umari, N. Vast, X. Wu, S. Baroni, *J. Phys.: Condens. Matter* **2017**, *29*, 465901.

[4] P. Giannozzi, S. Baroni, N. Bonini, M. Calandra, R. Car, C. Cavazzoni, D. Ceresoli, G. L. Chiarotti, M. Cococcioni, I. Dabo, A. D. Corso, S. de Gironcoli, S. Fabris, G. Fratesi, R. Gebauer, U. Gerstmann, C. Gougoussis, A. Kokalj, M. Lazzeri, L. Martin-Samos, N. Marzari, F. Mauri, R. Mazzarello, S. Paolini, A. Pasquarello, L. Paulatto, C. Sbraccia, S. Scandolo, G. Sclauzero, A. P. Seitsonen, A. Smogunov, P. Umari, R. M. Wentzcovitch, *J. Phys.: Condens. Matter* **2009**, *21*, 395502.

[5] M. J. van Setten, M. Giantomassi, E. Bousquet, M. J. Verstraete, D. R. Hamann, X. Gonze, G.-M. Rignanese, *Computer Physics Communications* **2018**, *226*, 39.

[6] D. R. Hamann, *Phys. Rev. B* **2013**, *88*, 085117.

[7] J. P. Perdew, A. Ruzsinszky, G. I. Csonka, O. A. Vydrov, G. E. Scuseria, L. A. Constantin, X. Zhou, K. Burke, *Phys. Rev. Lett.* **2008**, *100*, 136406.

[8] F. Birch, *Phys. Rev.* **1947**, *71*, 809.

[9] H. J. Monkhorst, J. D. Pack, *Phys. Rev. B* **1976**, *13*, 5188.

[10] M. Noh, S. Choi, D. Lee, M. Cho, C. Jeon, Y. Lee*, *New Physics: Sae Mulli* **2013**, *63*, 939.

[11] M. S. Paun, **n.d.**, 148.

[12] M. Yashima, R. Ali, *Solid State Ionics* **2009**, *180*, 120.

[13] L. Cao, E. Sozontov, J. Zegenhagen, *physica status solidi (a)* **2000**, *181*, 387.

[14] Z. Hou, K. Terakura, *J. Phys. Soc. Jpn.* **2010**, *79*, 114704.

[15] C. Ricca, I. Timrov, M. Cococcioni, N. Marzari, U. Aschauer, *Phys. Rev. Research* **2020**, *2*, 023313.

[16] A. Janotti, J. B. Varley, M. Choi, C. G. Van de Walle, *Physical Review B* **2014**, *90*, DOI 10.1103/PhysRevB.90.085202.

[17] R. Wahl, D. Vogtenhuber, G. Kresse, *Phys. Rev. B* **2008**, *78*, 104116.

[18] K. van Benthem, C. Elsässer, R. H. French, *Journal of Applied Physics* **2001**, *90*, 6156.

[19] S. H. Wemple, *Phys. Rev. B* **1970**, *2*, 2679.

[20] S. Yamanaka, M. Fujikane, T. Hamaguchi, H. Muta, T. Oyama, T. Matsuda, S. Kobayashi, K. Kurosaki, *Journal of Alloys and Compounds* **2003**, 5.

[21] T. Maekawa, K. Kurosaki, S. Yamanaka, *Journal of Alloys and Compounds* **2006**, *407*, 44.

[22] P. Umari, A. Pasquarello, *Physical Review Letters* **2002**, *89*, DOI 10.1103/PhysRevLett.89.157602.

[23] M. Cococcioni, S. de Gironcoli, *Phys. Rev. B* **2005**, *71*, 035105.

[24] M. Youssef, K. J. Van Vliet, B. Yildiz, *Physical Review Letters* **2017**, *119*, DOI 10.1103/PhysRevLett.119.126002.

[25] R. A. van der Berg, P. W. M. Blom, J. F. M. Cillessen, R. M. Wolf, *Appl. Phys. Lett.* **1995**, *66*, 697.
[26] M. Li, J. Li, L.-Q. Chen, B.-L. Gu, W. Duan, *Phys. Rev. B* **2015**, *92*, 115435.
[27] K. Klyukin, V. Alexandrov, *Physical Review B* **2017**, *95*, DOI 10.1103/PhysRevB.95.035301.
[28] L. L. Tao, J. Wang, *Journal of Applied Physics* **2016**, *119*, 224104.
[29] T. Shimada, T. Ueda, J. Wang, T. Kitamura, *Phys. Rev. B* **2013**, *87*, 174111.
[30] X. Liu, J. D. Burton, E. Y. Tsymbal, *Physical Review Letters* **2016**, *116*, DOI 10.1103/PhysRevLett.116.197602.
[31] "error opening file charge-density.dat in QE_6.3 (#105) · Issues · QEF - Quantum Espresso Foundation / q-e," can be found under https://gitlab.com/QEF/q-e/-/issues/105, **n.d.**
[32] "Should 'lelfield' with hybrid XC be disabled in QE 6.4.1? (#158) · Issues · QEF - Quantum Espresso Foundation / q-e," can be found under https://gitlab.com/QEF/q-e/-/issues/158, **n.d.**
[33] Li-Mo Wang, in *2006 25th International Conference on Microelectronics*, **2006**, pp. 576–579.
[34] T. Razzak, H. Chandrasekar, K. Hussain, C. H. Lee, A. Mamun, H. Xue, Z. Xia, S. H. Sohel, M. W. Rahman, S. Bajaj, C. Wang, W. Lu, A. Khan, S. Rajan, *Appl. Phys. Lett.* **2020**, *116*, 023507.
[35] J. W. Bennett, B. G. Hudson, I. K. Metz, D. Liang, S. Spurgeon, Q. Cui, S. E. Mason, *Computational Materials Science* **2019**, *170*, 109137.
[36] K. F. Garrity, J. W. Bennett, K. M. Rabe, D. Vanderbilt, *Computational Materials Science* **2014**, *81*, 446.
[37] D. Vanderbilt, *Phys. Rev. B* **1990**, *41*, 7892.
[38] B. Wang, W. Huang, L. Chi, M. Al-Hashimi, T. J. Marks, A. Facchetti, *Chemical Reviews* **2018**, *118*, 5690.
[39] N. Marzari, D. Vanderbilt, *Phys. Rev. B* **1997**, *56*, 12847.
[40] N. Marzari, A. A. Mostofi, J. R. Yates, I. Souza, D. Vanderbilt, *Rev. Mod. Phys.* **2012**, *84*, 1419.
[41] G. Pizzi, V. Vitale, R. Arita, S. Blügel, F. Freimuth, G. Géranton, M. Gibertini, D. Gresch, C. Johnson, T. Koretsune, J. Ibañez-Azpiroz, H. Lee, J.-M. Lihm, D. Marchand, A. Marrazzo, Y. Mokrousov, J. I. Mustafa, Y. Nohara, Y. Nomura, L. Paulatto, S. Poncé, T. Ponweiser, J. Qiao, F. Thöle, S. S. Tsirkin, M. Wierzbowska, N. Marzari, D. Vanderbilt, I. Souza, A. A. Mostofi, J. R. Yates, *J. Phys.: Condens. Matter* **2020**, *32*, 165902.
[42] D. G. Schlom, L.-Q. Chen, C.-B. Eom, K. M. Rabe, S. K. Streiffer, J.-M. Triscone, *Annual Review of Materials Research* **2007**, *37*, 589.
[43] A. Janotti, D. Steiauf, C. G. Van de Walle, *Physical Review B* **2011**, *84*, DOI 10.1103/PhysRevB.84.201304.
[44] J. E. Huheey, E. A. Keiter, R. L. Keiter, *Inorganic Chemistry : Principles of Structure and Reactivity*, New York, NY : HarperCollins College Publishers, **1993**.
